# Development and validation of a quantitative Proximity Extension Assay instrument with 21 proteins associated with cardiovascular risk (CVD-21)

**DOI:** 10.1371/journal.pone.0293465

**Published:** 2023-11-14

**Authors:** Agneta Siegbahn, Niclas Eriksson, Erika Assarsson, Martin Lundberg, Andrea Ballagi, Claes Held, Ralph A. H. Stewart, Harvey D. White, Mikael Åberg, Lars Wallentin

**Affiliations:** 1 Department of Medical Sciences, Clinical Chemistry, Uppsala University, Uppsala, Sweden; 2 Uppsala Clinical Research Center, Uppsala University, Uppsala, Sweden; 3 Science for Life Laboratory, Uppsala University, Uppsala, Sweden; 4 Olink Proteomics, Uppsala, Sweden; 5 Department of Medical Sciences, Cardiology, Uppsala University, Uppsala, Sweden; 6 Green Lane Cardiovascular Service, Te Whatu Ora Health New Zealand, Te Toka Tumai Auckland and University of Auckland, Auckland, New Zealand; University of Pisa, ITALY

## Abstract

**Background:**

Treatment of cardiovascular diseases (CVD) is a substantial burden to healthcare systems worldwide. New tools are needed to improve precision of treatment by optimizing the balance between efficacy, safety, and cost. We developed a high-throughput multi-marker decision support instrument which simultaneously quantifies proteins associated with CVD.

**Methods and findings:**

Candidate proteins independently associated with different clinical outcomes were selected from clinical studies by the screening of 368 circulating biomarkers. We then custom-designed a quantitative PEA-panel with 21 proteins (CVD-21) by including recombinant antigens as calibrator samples for normalization and absolute quantification of the proteins. The utility of the CVD-21 tool was evaluated in plasma samples from a case-control cohort of 4224 patients with chronic coronary syndrome (CCS) using multivariable Cox regression analyses and machine learning techniques. The assays in the CVD-21 tool gave good precision and high sensitivity with lower level of determination (LOD) between 0.03–0.7 pg/ml for five of the biomarkers. The dynamic range for the assays was sufficient to accurately quantify the biomarkers in the validation study except for troponin I, which in the modeling was replaced by high-sensitive cardiac troponin T (hs-TnT). We created seven different multimarker models, including a reference model with NT-proBNP, hs-TnT, GDF-15, IL-6, and cystatin C and one model with only clinical variables, for the comparison of the discriminative value of the CVD-21 tool. All models with biomarkers including hs-TnT provided similar discrimination for all outcomes, e.g. c-index between 0.68–0.86 and outperformed models using only clinical variables. Most important prognostic biomarkers were MMP-12, U-PAR, REN, VEGF-D, FGF-23, TFF3, ADM, and SCF.

**Conclusions:**

The CVD-21 tool is the very first instrument which with PEA simultaneously quantifies 21 proteins with associations to different CVD. Novel pathophysiologic and prognostic information beyond that of established biomarkers were identified by a number of proteins.

## Introduction

Cardiovascular disease (CVD) remains the most common cause of death worldwide despite a substantial decline in age-related mortality and morbidity during the last decades [1, 2]. Cardiovascular diseases are related to atherosclerosis, inflammation, and fibrosis in the coronary arteries and myocardium, processes which are associated with chronic coronary syndrome (CCS), acute coronary syndrome, myocardial infarction (MI), stroke, atrial fibrillation (AF), heart failure (HF) and sudden death [3, 4]. Novel modalities for treatment and prevention of these diseases have been available over the last decade including interventional procedures and lipid-lowering, antihypertensive, antithrombotic, anti-inflammatory, and HF medications. However, some of these new treatment strategies for these conditions have potential severe side-effects and add a substantial economic burden to society [3, 4]. Thus, there is a large need to develop and implement tools for better precision in obtaining the best balance between efficacy, safety, and cost when selecting treatment for the individual patient and also for monitoring the treatment effects over time [5, 6].

The levels of individual biomarkers together with clinical information have by us and others been shown to provide incremental information for diagnosis, prognostication, and decision support beyond clinical information in several cardiovascular diseases [5, 7–11]. By novel multiplex aptamer- and antibody-based technologies it is today possible to simultaneously screen a multitude of circulating biomarkers related to disease development, effects of treatment, and clinical outcomes [12–15]. One of these techniques is the Proximity Extension Assay (PEA). The technology is based on dual antibody recognition of the target and has over the last decade proven robust with high target specificity [12, 13, 16–18].

Given the possibility of protein biomarker screening in individual patients with good precision we aimed to utilize the PEA to create a quantitative multiplex CVD test for the precision medicine era. The foundation for the development of this test stems from our previous PEA screening of the relative importance of 368 proteins concerning associations with clinical outcomes in more than 10 000 patients with CAD or AF [19–22]. However, in the clinical setting information on biomarker levels needs to be based on precise and reproductive quantification rather than relative levels provided in the commercially available PEA panels. Therefore, we developed a multiplex protein panel allowing the simultaneous quantification of 21 circulating proteins which at previous PEA screening had independent and incremental importance for the prognostication of cardiovascular outcomes (MI, stroke, HF, bleeding, or cardiovascular death) in patients with different CVD. We herein describe the establishment of this tool and the evaluation of the prognostic value and utility of the candidate proteins in plasma samples from a large case:control cohort of patients with CCS.

## Material and methods

In the Stabilization of Atherosclerotic Plaque by Initiation of Darapladib Therapy (STABILITY) trial 15 828 patients with CCS were randomly assigned to receive either a once-daily dose of darapladib, a selective oral inhibitor of lipoprotein-associated phospholipase A_2_, or placebo [23, 24]. Patients were enrolled between December 2008 and April 2010 and the characteristics of all the patients included in the STABILITY trial have been presented in detail [24]. The STABILITY trial did not reach its primary target of superiority versus placebo concerning the primary composite endpoint major adverse cardiovascular events (MACE) (p = 0.20) but showed a statistically significant reduction (p<0.05) concerning major coronary events (MCE) hazard ratio, 0.90; 95% CI, 0.82 to 1.00; P = 0.045 [24]. All participants provided written informed consent, and the study was approved by all institutional ethics committees [23, 24].

Blood samples from the STABILITY trial were collected in EDTA tubes at randomization and immediately centrifuged. The plasma samples were frozen in aliquots and stored at -80°C until analysis.

Plasma samples from 4224 patients were included in the validation study of the proteomics (CVD-21) tool. This case-cohort study from the STABILITY included a random sample enriched with all patients with available biomarkers at baseline and with any of the 1351 MACE and 296 HF events recorded during 3 years follow-up in the 13,164 patients in the STABILITY biomarker substudy, i.e., all cases with any cardiovascular event and approximately two times as many non-cases from the same cohort. Definitions of all fatal and ischemic outcome events (MACE, MCE, MI, stroke) in the STABILITY cohort were pre-specified and these events were adjudicated by an independent clinical events committee. Heart failure events were recorded as readmission at hospital with a diagnosis of HF [23, 24].

### Gold standard immunoassays

Growth differentiating factor -15 (GDF-15), N-terminal prohormone of brain natriuretic peptide (NT-pro-BNP), high-sensitivity Troponin T (cTnT-hs), and Cystatin C concentrations were analyzed by Roche electrochemiluminescence (ECL) immunoassays on a Cobas Analytics e601 instrument (Roche Diagnostics). Interleukin-6 was analyzed by Quantikine ^®^ HS Human IL-6 Immunoassay, R&D Systems, on a Tecan Freedom EVOlyzer. All analyses in the STABILITY biomarker substudy were done according to the instructions of the manufacturer at the Uppsala Clinical Research Center (UCR) Laboratory, Uppsala University, Uppsala, Sweden and are previously published [9, 25, 26]. The biomarker results previously obtained with the conventional assays in plasma from patients included in the case-cohort validation study were used in comparison with the results analyzed with the quantitative CVD-21 PEA tool and for the validation of the CVD-21 tool in patients with CCS.

### Multiplex proteomic analysis, Proximity Extension Assay

With the predesigned Olink® Target 96 panels, PEA multiplex technology is used with 92 oligonucleotide-labelled antibody pairs (PEA probes) that can bind their respective target present in the sample. Upon proximity binding of their specific antigens and DNA polymerase-aided joining of the paired oligonucleotides, new DNA amplicons are formed and each individual DNA sequence is then detected and quantified using specific primers by microfluidic qPCR using Fluidigm Biomark^TM^ HD. The resulting Ct values are normalized with help of internal and external controls to generate Normalized Protein eXpression (NPX) data correlating with the protein concentration in the sample. Lower level of detection (LOD) is defined as negative control plus 3 standard deviations (SD) [13]. A complete analysis of a PEA panel takes 24 hours. The PEA analyses were performed at the Clinical Biomarkers Facility (now called Affinity Proteomics), Science for Life Laboratory, Uppsala University, Uppsala, Sweden.

Reagents for the CVD-21 tool are presented in S1 File.

### Normalization and absolute quantification of biomarkers in the CVD-21 panel

Each run included triplicate measurements of four different calibrators (High, Middle, Low, and Blank) for normalization and absolute quantification, as well as two control sample pools, QC1 and QC2, for precision calculations. For each sample and data point, the corresponding Ct-value for the internal Extension control was subtracted, thereby normalizing for technical variation within one run. Normalization between runs was then performed for each assay by subtracting the corresponding dCt-value for the calibrator (median of each calibrator triplicate and mean of the three different calibrators) from the dCt-values generated. In the final step of the pre-processing procedure the values are set relative to a correction factor determined by Olink Proteomics. The resulting NPX unit is on a log2 scale where a larger number represents a higher protein level in the sample, typically with the background level at around zero.

Standard curves were generated in multiplex for all assays included in the CVD-21 panel, using recombinant antigens for all 21 assays. The curves were analyzed as duplicate measurements in two consecutive experiments. Data were normalized and a four-parameter logistic regression (4-PL) curve fitting (Extreme Optimization™) was applied to all data points (2 runs, 2 replicates and 30 concentrations at 2-fold dilutions) and used to determine LOD, lower and upper limit of quantification (LLOQ and ULOQ) for each assay. LLOQ and ULOQ were defined by accuracy and precision ≤30%. As the exact quantification of the antigen standards being used to develop calibrator samples/standard curves will vary between different vendors, different assay platforms might report somewhat different concentration values. We therefore calibrated five assays to clinically well-validated immunoassays (hs-IL-6 Elisa R&D systems, GDF-15, cystatin C, NT-pro-BNP, and troponin T by Elecsys commercial kits, Roche Diagnostics). This was done using data, within LOQ, from 40 samples that were previously analyzed with the respective reference methods. The input concentrations to the standard curves for those five assays were value reassigned to best fit the clinically used assays. After that the 4-PL was remade on the adjusted standard curves.

### Linearity of dilution

In the current 21-plex protocol, native samples were analyzed so that linearity was studied under true matrix conditions. This was done by mixing a sample containing relatively high endogenous concentration of the protein analyte with a sample containing a low concentration at different ratios, to give five equally spaced concentrations. Native samples were chosen to give as wide a range as possible, requiring several different sample combinations to be included in the test, all depending on the endogenous concentrations. A ten-fold dilution was applied to all samples and the standard protocol was run to quantify all samples. For each intermediate sample, the theoretical concentration was calculated for each protein using the measured concentrations of the high and low samples (from each sample set).

### Statistics

#### Accuracy and intra-assay precision of the assays in the CVD-21 tool analyzed in the STABILITY validation study

The accuracy and intra-assay coefficient of variation (CV) were calculated for all assays per QC1 and QC2 values for each run by the mean of the 6 QC replicates divided by the reference value and the standard deviation of the 6 QC replicates divided by the mean, respectively. The validation study included 28 chips with 2 plates per chip. Thus, the calculations were recorded from 56 plates with QC1 and QC2 analyzed in triplicate.

#### Comparisons of CVD-21 quantitative PEA versus gold standard immunoassays

Comparisons between CVD-21 assays and gold standard immunoassays, both on log2 scale, were presented visually by scatter-plots and Bland-Altman plots. The Spearman correlation and linear regression r^2^ were presented within the scatter-plot and mean, standard deviation and confidence limits presented within the Bland-Altman plot.

#### Clinical outcome analyses

Missing values on the CVD-21 markers were imputed using a lognormal distribution as implemented in the R-package lnormimp using the minimum observed value for imputation below LLOQ and the Olink supplied ULOQ value for imputation above ULOQ. The following number of values (described as marker (number imputed below LLOQ, number imputed above ULOQ)) were imputed for the CVD-21 biomarkers; CST3 (0, 65), FGF-23 (3, 0), GDF-15 (0, 2), HGF (1, 0), KIM1 (2, 0), MMP-12 (1, 0), OPG (1, 0), REN (0, 12), TFF3 (1, 1), TNNI3 (2, 0), U-PAR (1, 0) and VEGF-D (2, 0). Missing values on biomarkers other than CVD-21 and the covariates BMI and smoking status were imputed using one round of multiple imputation as implemented in the R-package mice. The number of missing values that were imputed were; BMI (9), Smoking (24), Cystatin-C (5), GDF-15 (8), IL-6 (3), NT-proBNP (3) and Troponin-T (130).

Time-to-event outcomes were analyzed using multiple Cox-regression models. The models included the sampling weights according to the study design and standard errors were calculated using a robust-sandwich estimator. All continuous variables were modeled using a four knot restricted cubic spline (knots placed at 5^th^, 35^th^, 65^th^, and 95^th^ percentiles) with corresponding hazard ratios presented for the third vs. the first quartile level of the distribution. Harrell’s C-statistic was used to quantify the predictive performance of each variable or model [27].

Predictive performance of different combinations of variables was evaluated using multiple Cox-regression models (including sampling weights and robust standard errors) and random survival forest models. We constructed seven different models that were used in the multiplex statistical analysis; 1. A clinical model: age, sex, BMI, smoking, diabetes mellitus (DM), hypertension, previous peripheral artery disease (PAD), prior MI or stroke, congestive HF, previous PCI or CABG. 2. The CVD-21 model; all panel biomarkers. 3. A laboratory model: NT-proBNP, hs-cTnT, GDF-15, IL-6, Cystatin C analyzed by established immunoassays. 4. A combined model including variables from model 2 and 3 with 5 markers analyzed by established immunoassays. 5. CVD-21; plus model; all CVD-21 biomarkers except troponin I which was changed to troponin T analyzed by established immunoassays. 6. ABC CVD-21 model; model 5 + the clinical model. 7. ABC Combined model; model 4+ clinical model.

For these multiple Cox-regression models, where focus was on prediction, we applied an approach where we first fitted a model with all continuous variables modeled as four knot splines. Based on each variable’s χ^2^ statistic from the initial model we selected which continuous variables to prioritize for nonlinear estimation using a four knot spline whereas the others were modeled as linear. The purpose of this step was to lower the total degrees of freedom in the model which in turn reduced the amount of overfitting. The fraction of new information (FNI) per variable in each model, which is the proportion of total predictive information added by each variable, was calculated as 1 –(modA likelihood ratio χ^2^ / modB likelihood ratio χ^2^) where modA is a model without the biomarker of interest and modB is the full model [28]. Each model was internally validated using 300 bootstrap samples (function validate from the R-package rms) and the bias adjusted Harrell’s C-statistics are presented. Pairwise comparisons between the performance of the different models in terms of C-statistic were done using a method for comparing two correlated C indices for survival outcomes by Kang et al. [29] implemented in the R-package compareC.

The random survival models were fitted using the R-package ranger with the following settings: number of trees = 10000, variable importance mode = permutation, mtry (randomly selected number of variables to possibly split in each node) = square root of total number of variables, minimal node size = 3, and splitrule = maxstat (maximally selected rank statistics). Harrell’s C-statistic on the out of bag predictions was reported [30, 31].

All analyses were performed using R version 4.1.2

## Results

### Construction and design of the quantitative CVD-21 PEA panel

**Fig 1:**
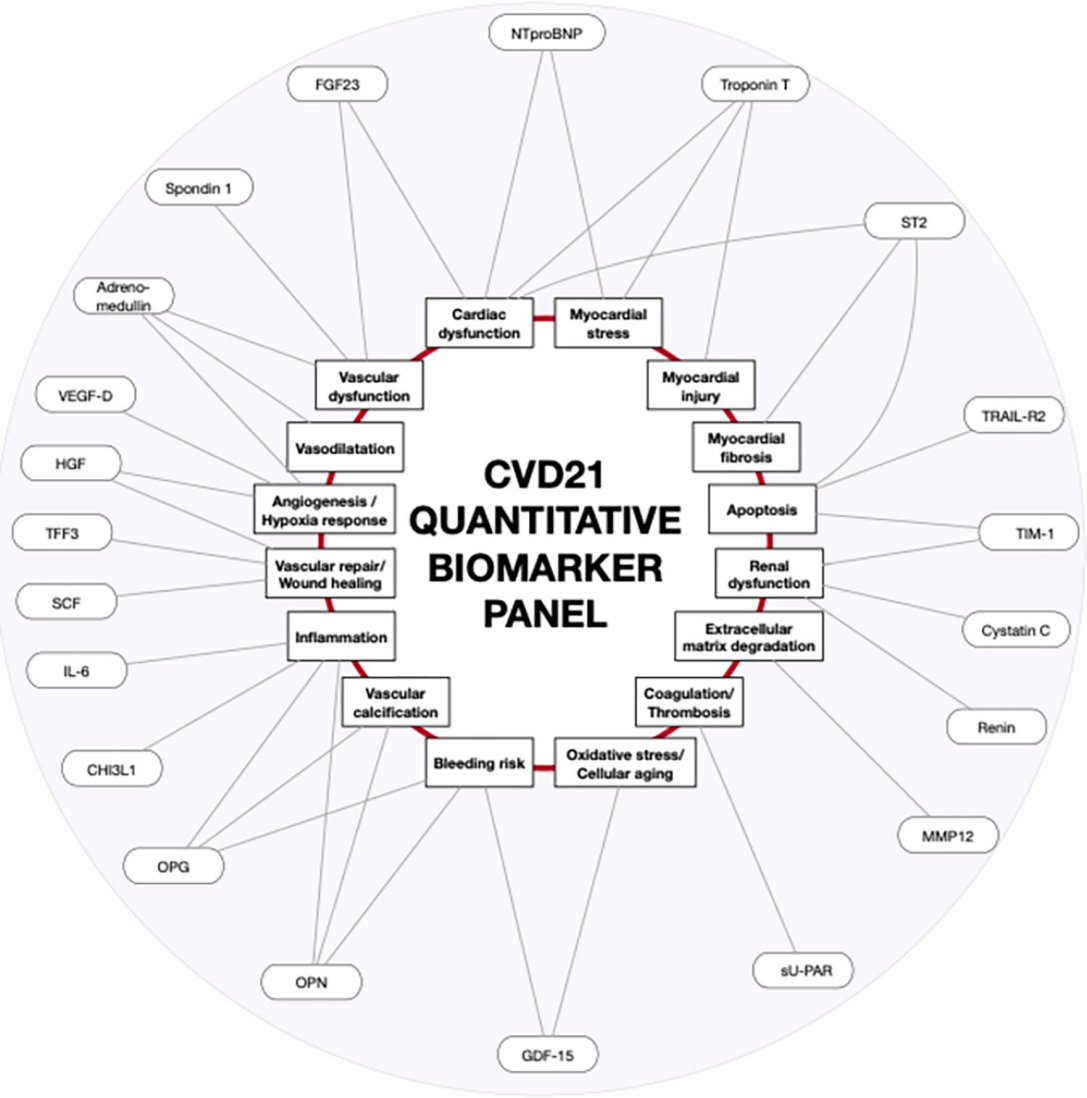
Illustration of the CVD-21 multiplex quantitative biomarker panel and the individual pathways and their connections to the development of cardiovascular and bleeding events. Abbreviations: CHI3L1 (chitinase-3 like protein, also called YKL-40 (heparin -and chitin-binding glycoprotein), FGF23 (fibroblast growth factor 23), GDF-15 (growth differentiation factor 15), HGF (hepatocyte growth factor), IL-6 (interleukin-6), MMP12 (metalloproteinase-12), NTproBNP (N-terminal prohormone of brain natriuretic peptide), OPG (osteoprotegerin), OPN (osteopontin), SCF (stem cell factor), ST2 (suppression of tumorigenicity), sU-PAR (soluble urokinase-type plasminogen activator receptor), TFF3 (trefoil factor 3), TIM-1/KIM1 (T-cell immunoglobulin and mucin domain-containing protein), TRAIL-R2 (tumor necrosis factor (TNF)-related apoptosis-inducing ligand 2), VEGF-D (vascular endothelial growth factor -D).

In order to identify additional proteins and protein patterns associated with clinical events and underlying pathophysiological processes in patients with CAD or AF we performed multiplex protein screening analyses using the Olink ® Target 96 CVD I, CVD II, CVD III and Inflammation panels on 10 622 patient samples from the STABILITY and LURIC (Ludwigshafen Risk and Cardiovascular Health) studies and from the ARISTOTLE and RELY trials [19–22]. Based on the statistical associations with different outcomes in these screening projects, we selected 21 proteins with statistically significant and independent associations with CVD events and combined them into a quantitative PEA panel, CVD-21. As illustrated in **Fig 1**, the proteins included in the CVD-21 panel reflect a large number of pathophysiological processes associated with cardiovascular diseases.

**S1 Table** shows the biomarkers that are included in the CVD-21 panel and in which Target 96 panel they are included.

### Analytical validation of the CVD-21 panel

#### Sensitivity

**Fig 2:**
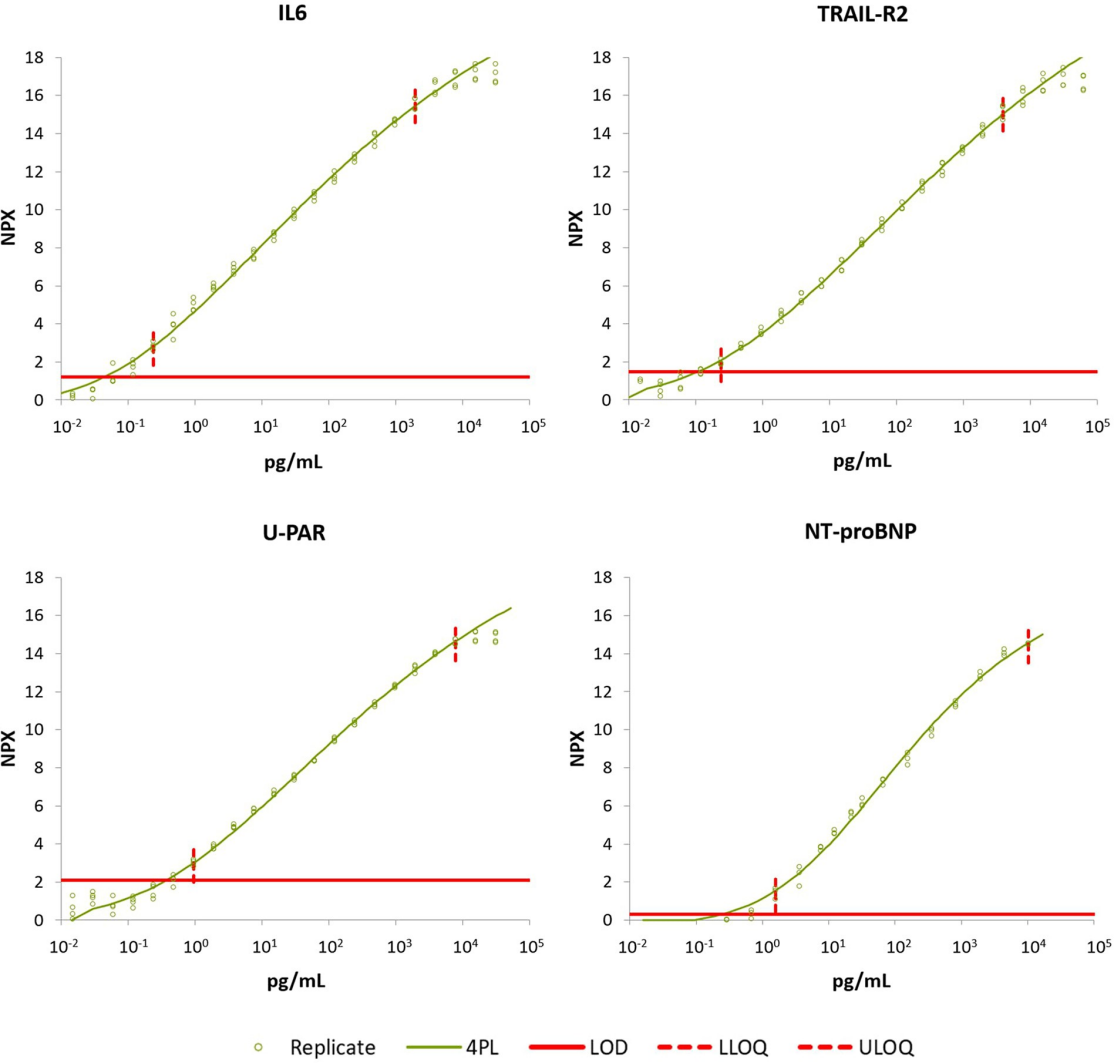
Antigen standard curves for four of the assays in the CVD-21 panel. Antigen standard curves were generated for all 21 assays and analyzed in multiplex. The figure shows the standard curves for IL-6, TRAIL-R2, U-PAR, and NT-proBNP. Data were normalized against an internal control assay and calibrators, and curve-fitting was performed using 4-PL. Circles show the triplicate data points from two experiments. Lines show the fitted curve. Abbreviations: IL6 (interleukin-6), TRAIL-R2 (tumor necrosis factor (TNF)-related apoptosis-inducing ligand 2), U-PAR (soluble urokinase-type plasminogen activator receptor), NT-proBNP (N-terminal prohormone of brain natriuretic peptide), NPX (normalized protein expression), LOD (level of determination), LLOQ (lower level of quantification), ULOQ (upper level of quantification).

For all assays in the CVD-21 panel, standard curves were generated in multiplex using recombinant antigens. **Fig 2** shows the standard curves for IL-6, NT-proBNP, U-PAR, and tumor necrosis factor (TNF)-related apoptosis-inducing ligand 2 (TRAIL-R2) and **S2 Table** displays the analysis summary of the sensitivity parameters for all the included biomarkers. The assays gave precise measurements and high sensitivity. The most sensitive assay was IL-6 with LOD of 0.03 pg/ml and LLOQ of 0.1 pg/ml. Very high sensitivity with LOD < 1 pg/ml was also recorded for the assays of NT-proBNP, U-PAR, and TRAIL-R2 (**S2 Table)**.

#### Linearity of dilution and relative error of the CVD-21 assays

The linearity of the CVD-21 assays was tested as previously described and **S1 Fig** shows the results for four assays, IL-6, TRAIL-R2, U-PAR, and NT-proBNP and **S3 Table** presents all assays’ average CV percent and maximum and average relative error. All assays showed excellent linearity. The maximal absolute relative error was below 20% and average relative error was very good with all assays at 7% or below, except for NT-proBNP. The average sample-in-sample linearity study of the NT-proBNP assay indicated an average relative error of -14% over four sample sets with three data points each (relative error defined as [measured-theoretical]/[theoretical] concentration). The worst data point was -39% and is displayed in **S1 Fig and S3 Table**. This relatively poor relative error could be caused by imprecise measurement or dilution of the ‘high’ sample, or that the linearity at the higher end of the measuring range is suboptimal.

The linearity study of all assays demonstrated that the chosen approach for absolute quantification performs well for all assays included in the CVD-21 panel.

#### Dynamic range

**Fig 3:**
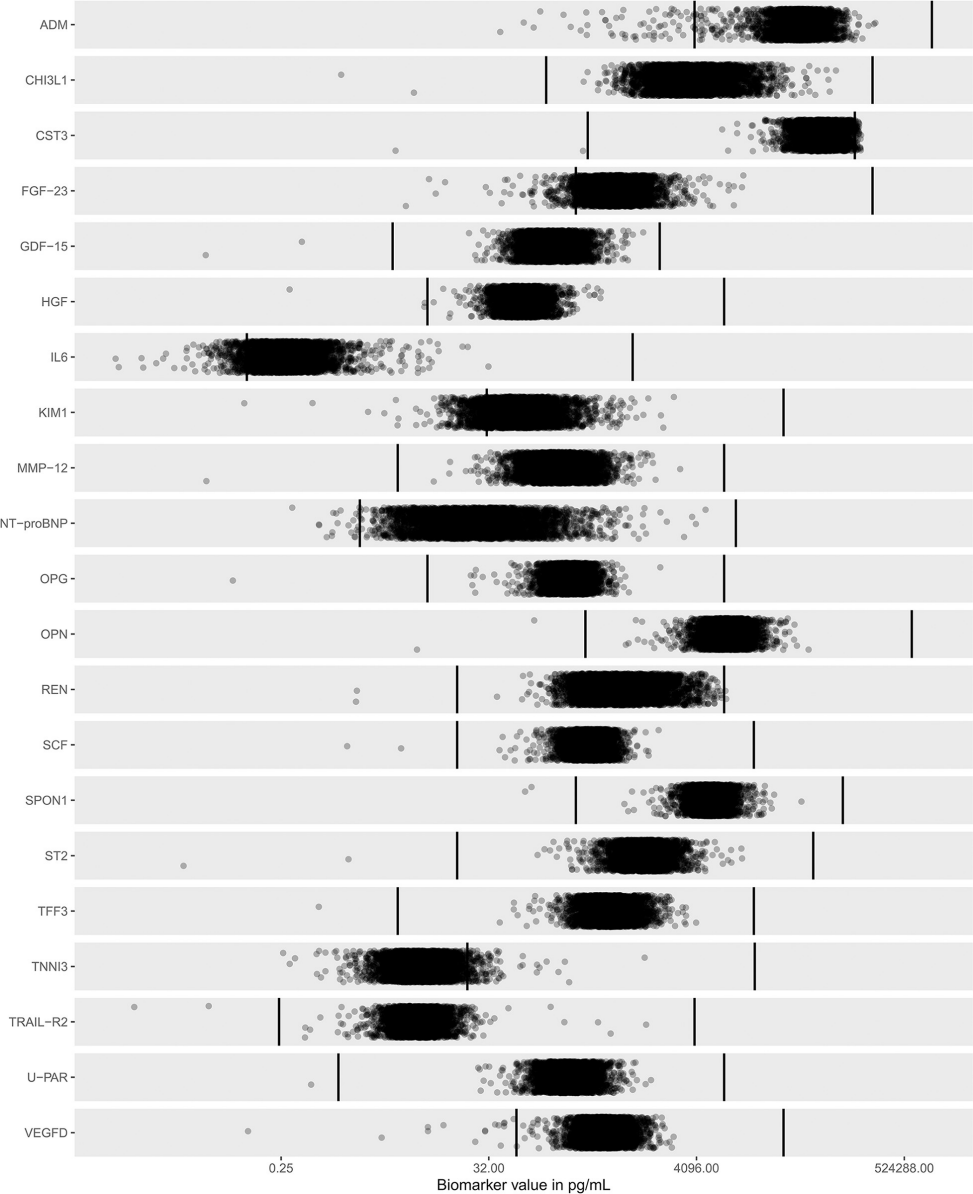
Dynamic range and sample distribution for all assays of 4224 samples from the STABILITY study. The vertical lines represent the LLOQ and ULOQ per assay derived from the sensitivity experiments. Circles show concentrations of the plasma samples. All plasma samples were prediluted 1:10. Abbreviations: ADM (adrenomedullin), CHI3L1 (chitinase-3 like protein, also called YKL-40 (heparin -and chitin-binding glycoprotein), CST3 (cystatin C), FGF-23 (fibroblast growth factor 23), GDF-15 (growth differentiation factor 15), HGF (hepatocyte growth factor), IL-6 (interleukin-6), TIM1/KIM1 (T-cell immunoglobulin and mucin domain-containing protein), MMP12 (metalloproteinase-12), NT-proBNP (N-terminal prohormone of brain natriuretic peptide), OPG (osteoprotegerin), OPN (osteopontin), REN (renin), SCF (stem cell factor), SPON1 (spondin-1), ST2 (suppression of tumorigenicity 2), TFF3 (trefoil factor 3), TRAIL-R2 (tumor necrosis factor (TNF)-related apoptosis-inducing ligand 2), TNNI3 (troponin I), U-PAR (soluble urokinase-type plasminogen activator receptor), VEGF-D (vascular endothelial growth factor -D).

4224 samples, thawed twice, from patients with CAD included in the STABILITY study were analyzed using the CVD-21 panel and plotted within LLOQ and ULOQ. The distribution is shown in **Fig 3 and S4 Table**. The dynamic range for the different assays covered most samples from this large study and only IL-6 and TIM-1/KIM1 (T-cell immunoglobulin and mucin domain-containing protein) had more than 5% of the sample values below LLOQ, 5.8% and 11.8% respectively. VEGF-D (vascular endothelial growth factor-D), NT-proBNP, FGF-23 and ADM (adrenomedullin) displayed values between 0.4–4% below LLOQ. IL-6, NT-proBNP, U-PAR, and TRAIL-R2 had the widest dynamic range, log 10 range from 3.8–4.2 (**S2 Table)**. However, 97% of the levels recorded for troponin I were below the LLOQ, mainly caused by the ten-fold predilution, which shows that this assay, in its current state, was not suitable for use in clinical settings. Only cystatin C had 0.6% of the levels above the ULOQ. Thus, the CVD-21 assays, except troponin I, were found to accurately quantify 20 selected biomarkers in plasma from a clinical setting of patients with cardiovascular disease.

#### Precision of the CVD-21 assays analyzed by QC1 and QC2 in the STABILITY validation study

**Fig 4:**
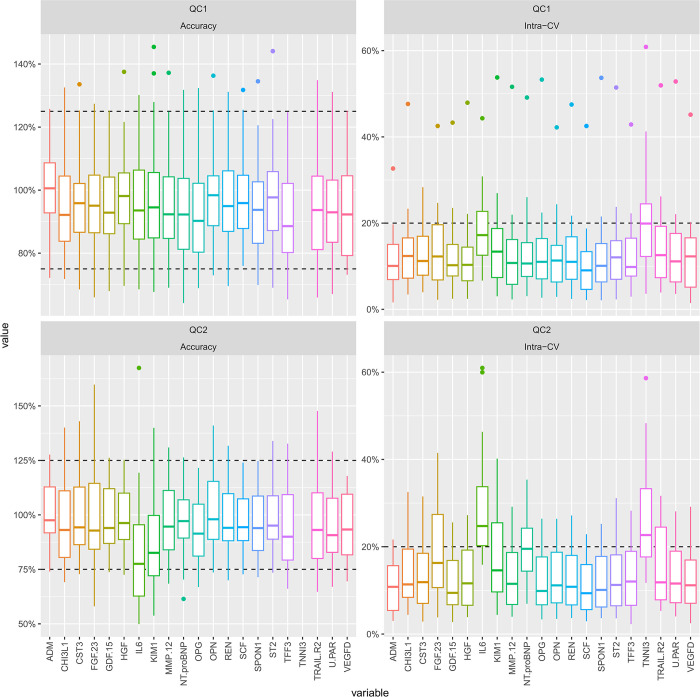
Box plots of accuracy and intraassay precision of the CVD-21 assays determined from QC1 and QC2 included on 28 runs including 56 plates. Accuracy and intra-assay coefficient of variation (CV)% were calculated per QC1 and QC2 by the mean of 6 replicates and divided by the reference value and the SD of the 6 replicates divided by the mean, respectively. Each boxplot represents the lower quartile, median, and upper quartile and the whiskers extend to the lowest and highest value within 1.5 times the interquartile range outside of the box. Outliers outside of the range of the whiskers are marked with a dot. Abbreviations: ADM (adrenomedullin), CHI3L1 (chitinase-3 like protein, also called YKL-40 (heparin -and chitin-binding glycoprotein), CST3 (cystatin C), FGF-23 (fibroblast growth factor 23), GDF-15 (growth differentiation factor 15), HGF (hepatocyte growth factor), IL6 (interleukin-6), TIM1/KIM1 (T-cell immunoglobulin and mucin domain-containing protein), MMP12 (metalloproteinase-12), NT-proBNP (N-terminal prohormone of brain natriuretic peptide), OPG (osteoprotegerin), OPN (osteopontin), REN (renin), SCF (stem cell factor), SPON1 (spondin-1), ST2 (suppression of tumorigenicity 2), TFF3 (trefoil factor 3), TNNI3 (troponin I), TRAIL-R2 (tumor necrosis factor (TNF)-related apoptosis-inducing ligand 2), U-PAR (soluble urokinase-type plasminogen activator receptor), VEGF-D (vascular endothelial growth factor -D).

We used QC1 and QC2 analyzed in triplicate in 56 plates for the calculations of accuracy and precision in the validation study. **Fig 4 and S5 Table** show that all assays, except for troponin-I, were within acceptance criteria, with values ranging between 75–125%. The average intra-CV was below 20% for all assays except for IL-6 measured in QC2. This can be explained by the low reference value of IL-6, which after predilution 1:10 is 0.13 pg/ml, that is close to the LLOQ, 0.1 pg/ml, in this pool of samples from healthy individuals.

Except for troponin-I the evaluation shows good precision and accuracy for the assays in the CVD-21 panel. The concentrations of the biomarkers in the control samples QC1 and QC2 can be used for precision calculations in future runs (**S6 Table**).

#### Comparison between golden standard immunoassays and quantitative PEA measurements

**Fig 5:**
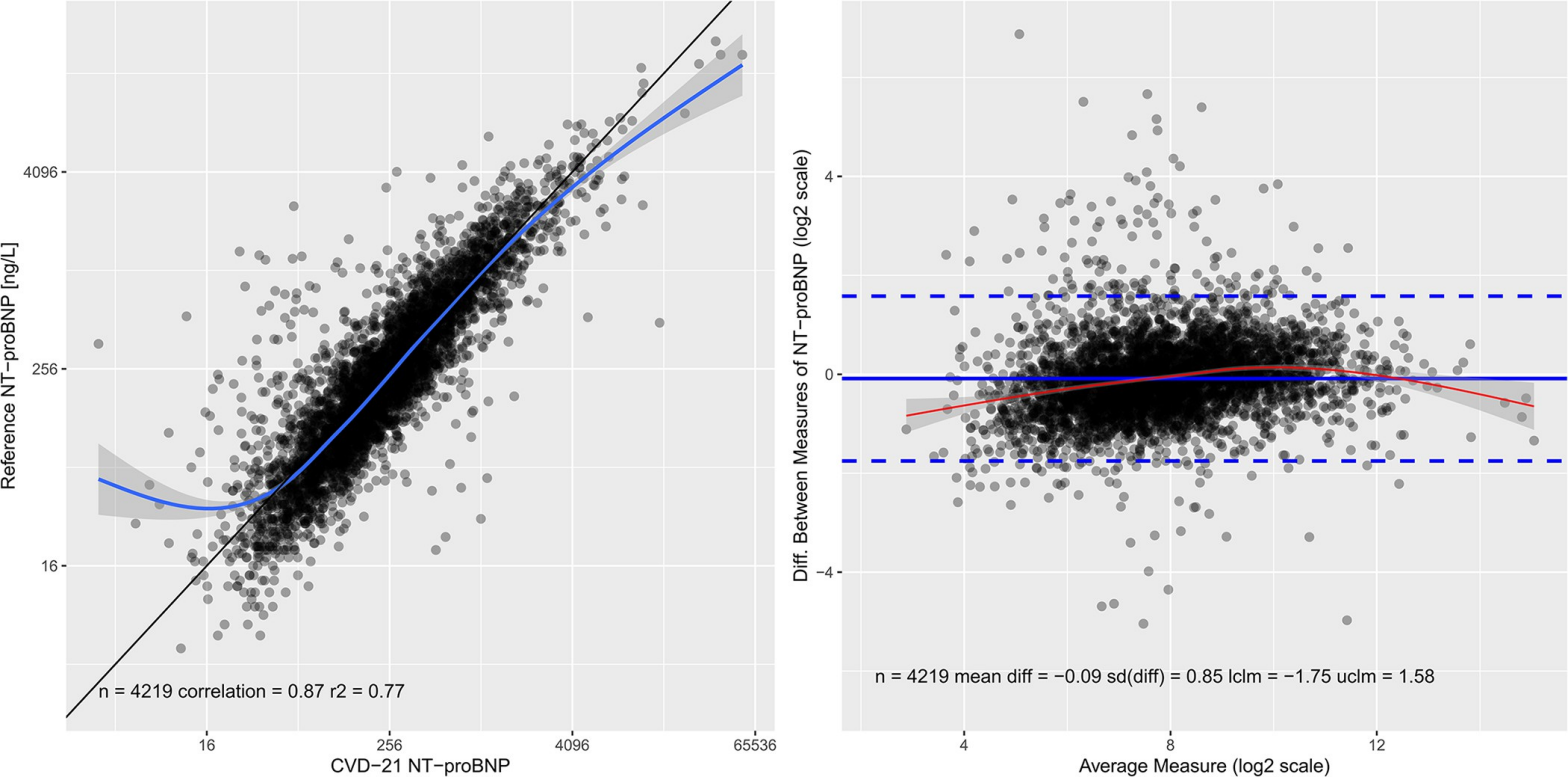
Scatter plot and Bland-Altman comparison between NT-proBNP measured by a golden standard method and CVD-21 quantitative PEA. Scatter plot (left) and Bland-Altman plot (right) for the comparison of NT-proBNP. The unit for the ECL immunoassay measured NT-proBNP is ng/L and the CVD-21 measurements have been multiplied by a factor of 10 to be on the same unit. The scatter plot includes a black identity line and a blue smoothed line (with 95% confidence interval in gray). The Bland-Altman plot is based on log2 transformed values of the biomarkers and it includes a solid blue line for the average, dotted blue lines for the confidence limits, and a red line showing a smoothed line (with 95% confidence interval in gray). NT-proBNP (N-terminal prohormone of brain natriuretic peptide).

To further validate the quantitative PEA assays, a comparison between the ECL (Roche Diagnostics) and ELISA methods and PEA was performed. **Fig 5** shows the comparison for NT-proBNP. A Spearman correlation 0.87 and R^2^ = 0.77 was observed between NT-proBNP concentrations obtained by the two methods. A Bland-Altman diagram indicated similar results **(Fig 5)**. Also, the GDF-15 assay had good correlation with the ECL method (0.80 R^2^ = 0.64), while the correlation was weaker for IL-6 (0.72 R^2^ = 0.52) and cystatin C (0.64 R^2^ = 0.41). The correlation of all the biomarkers in the CVD-21 panel is shown in **S2 Fig.**

However, the CVD-21 troponin-I was not possible to evaluate due to the low sensitivity of the assay.

### Validation of the CVD-21 tool in patients with chronic coronary syndrome (CCS)

The case-cohort study from STABILITY included all cases from the biomarker substudy with available biomarkers at baseline and any cardiovascular event and two times as many non-cases from the same cohort. During the median follow-up of 3.7 years there were 1351 MACE, 1163 MCE, 612 cardiovascular deaths, 652 MI, 296 HF, 831 cardiovascular deaths or hospitalization for HF, and 267 strokes. The clinical characteristics and the biomarker levels in the cases with different events and the corresponding random cohorts without events are shown in **S7 and S8 Tables**.

#### Estimates of effect and variable importance for the predictors in the ABC CVD-21 model for the outcomes MACE, MCE, MI, and cardiovascular death or hospitalization for HF

**Fig 6:**
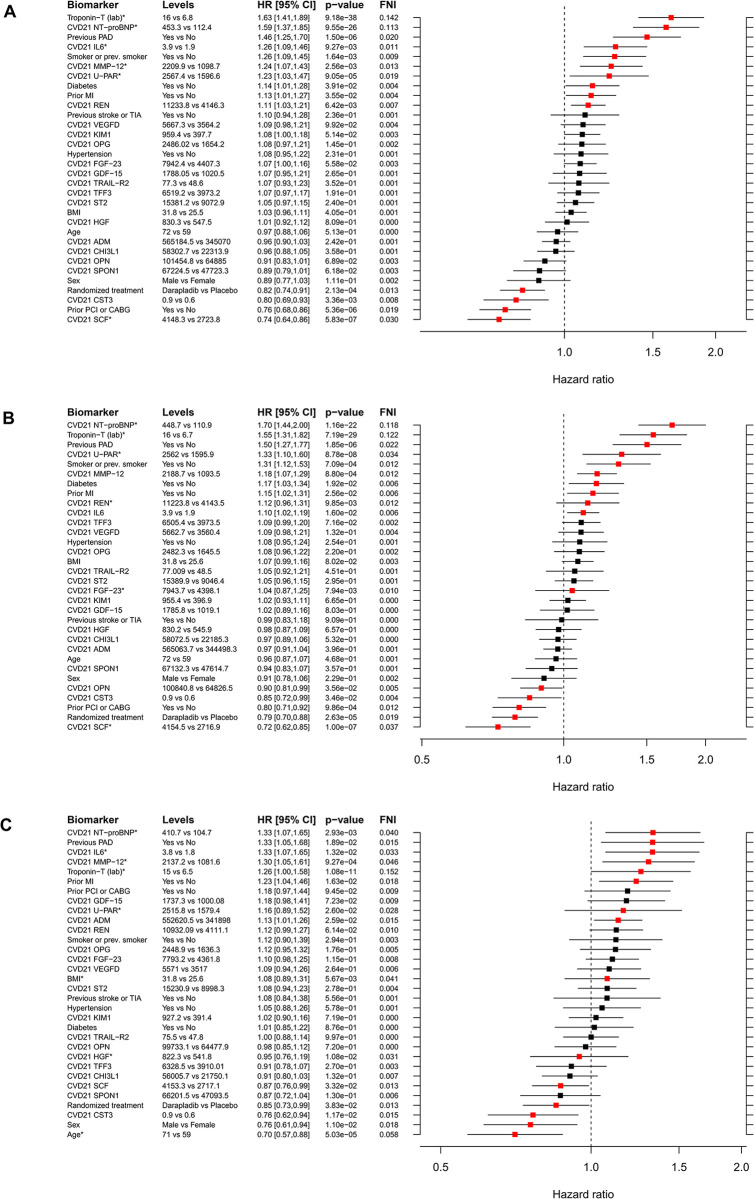
Forest plot for the predictors from the ABC CVD-21 model for the outcomes A) MACE, B) MCE and C) MI. Note that variables marked with * are modeled using a four knot spline and have a significant overall effect (the confidence interval could overlap 1). Abbreviations: ADM (adrenomedullin), CABG (coronary artery bypass graft), CHI3L1 (chitinase-3 like protein, also called YKL-40 (heparin -and chitin-binding glycoprotein), CST3 (cystatin C), FGF-23 (fibroblast growth factor 23), GDF-15 (growth differentiation factor 15), HGF (hepatocyte growth factor), IL-6 (interleukin-6), TIM1/KIM1 (T-cell immunoglobulin and mucin domain-containing protein), MI (myocardial infarction), MMP12 (metalloproteinase-12), NT-Pro-BNP (N-terminal prohormone of brain natriuretic peptide), OPG (osteoprotegerin), OPN (osteopontin), PCI (percutaneous coronary intervention), REN (renin), SCF (stem cell factor), SPON1 (spondin-1), ST2 (suppression of tumorigenicity 2), TFF3 (trefoil factor 3), TIA (transient ischemic attack), TNNI3 (troponin I), TRAIL-R2 (tumor necrosis factor (TNF)-related apoptosis-inducing ligand 2), U-PAR (soluble urokinase-type plasminogen activator receptor), VEGF-D (vascular endothelial growth factor -D).

We created seven different multiplex models for the evaluation and comparison of associations and incremental prognostic values of the CVD-21 biomarker tool in patients with CCS (see description in the statistical methods section). The hazard ratio and variable importance for each predictor in the ABC CVD-21 model for the outcomes of MACE, MCE, MI, and cardiovascular death or hospitalization of HF is illustrated in **Fig 6A–6C and S3 Fig**. The prognostically most important (FNI>0.01) biomarkers for MACE in the CVD-21 model were hs-TnT, NT-proBNP, IL-6, metalloproteinase-12 (MMP-12), U-PAR associated with increased risk and stem cell factor (SCF) associated with reduced risk, **Fig 6A.** In these models the fraction of novel prognostic information (FNI) contributed by hs-TnT, NT-proBNP was in the magnitude of 0.10–0.15 while clinical variables and other biomarkers in the model contributed less, i.e., < 0.05.

The individual variable importance for MCE is shown in **Fig 6B.** NT-proBNP, Troponin T, IL-6, U-PAR, MMP-12, and renin (REN), were the most important (FNI >0.01) biomarkers associated with increased risk and again SCF with reduced risk.

For MI the prognostically most important biomarkers included in the ABC CVD-21 model are shown in **Fig 6C**. hs-cTnT measured by the ECL immunoassay was the significantly most important biomarker for increased risk which had an FNI >0.01 together with MMP-12, NT-proBNP, IL-6, U-PAR, ADM, while SCF was associated with reduced risk.

Finally, the most important biomarkers (FNI >0.01) for cardiovascular death or hospitalization for HF were conveyed by NT-proBNP, hs-TnT, trefoil factor 3 (TFF3), VEGF-D, IL-6, and REN while SCF, ADM, and osteopontin (OPN), were associated with reduced risk.

Concerning clinical variables consistently associated with increased risk (FNI 0.01–0.03) in these models were previous PAD and smoking. Randomized treatment and previous PCI or CABG were consistently associated with reduced risk **(Fig 6, S3 Fig)**.

#### Prognostic values of the different multiplex models of all outcomes recorded in the STABILITY study

**Fig 7:**
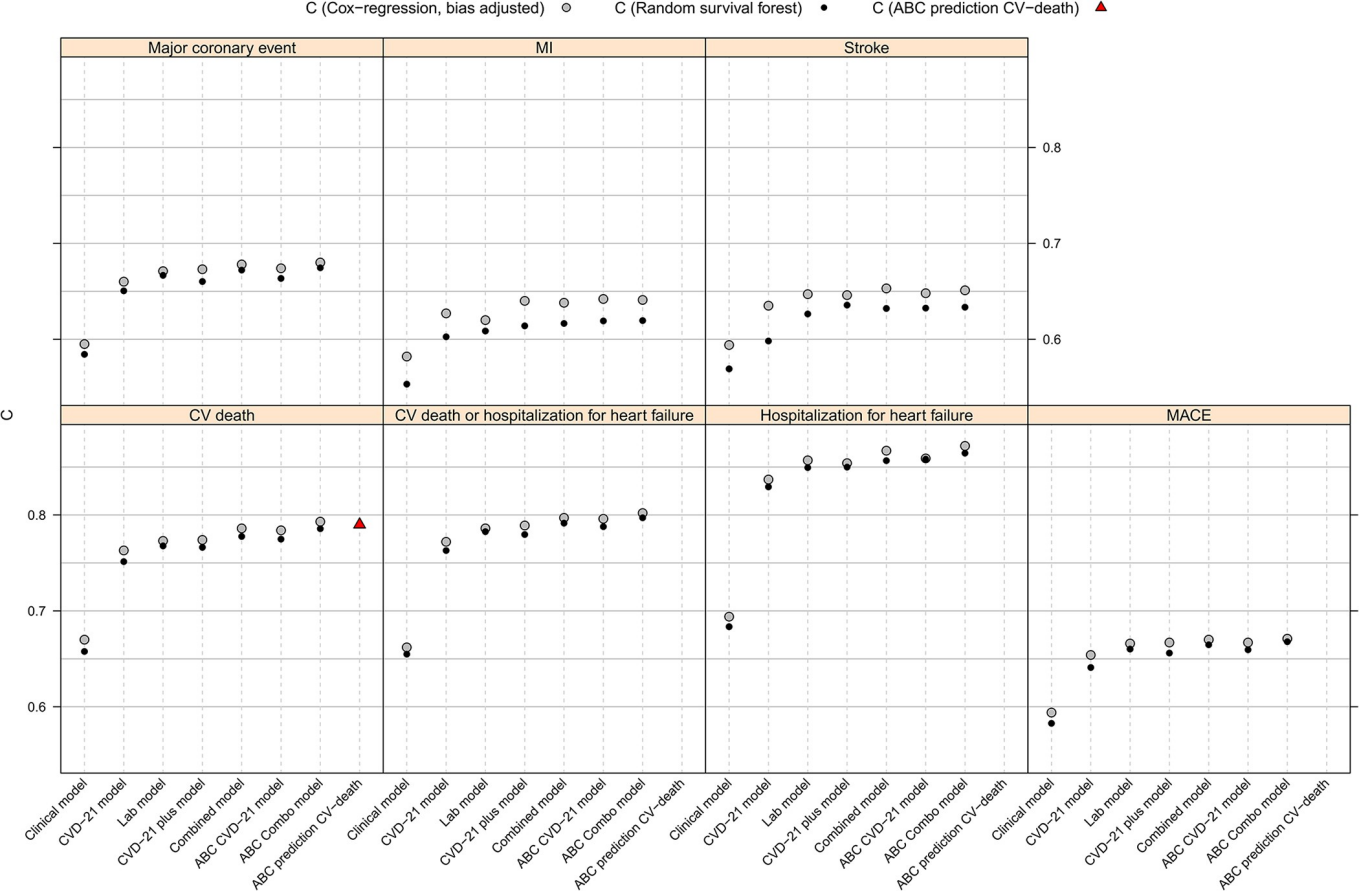
Summary of Cox-regression and random survival forest analyses of multiple model; summary of estimated C-indices for the different models and outcomes. Abbreviations: MI (myocardial infarction, CV (cardiovascular), MACE (major adverse cardiovascular event).

We used the seven different multiplex models to compare the prognostic value for all outcome events in the STABILITY study. The PEA assay for troponin I did not quantify more than 3% of the samples in the validation study. Therefore, we decided to evaluate the CVD-21 tool with both troponin I and hs-cTnT. The previously presented ABC-CHD risk score for prevention of death in patients with chronic CHD [9] includes age, NT-proBNP, hs-cTnT, LDL-cholesterol, and clinical variables and was also used for the comparison of prediction values for the outcomes of cardiovascular death. **Fig 7** shows that the laboratory model and the CVD-21plus model had similar c-indices for all outcomes. The ABC CVD-21 model achieved c-indices of the same magnitude as the combined and ABC combined models for all the recorded events. The ABC CVD-21 model achieved c-index 0.67 for MCE, 0.80 for cardiovascular death or hospitalization for heart failure and 0.87 for heart failure. These five models included hs-cTnT measured by the ECL immunoassay with very high sensitivity. The CVD-21 model achieved slightly lower c-indices for all events, from 0.63 for MI to 0.66 for MCE and 0.83 for HF, clearly showing that this was dependent on the inadequate sensitivity of troponin I **(Fig 3).** For comparison, for CV-death the ABC-CHD risk score achieved similar c-index as the CVD-21 models that included hs-cTnT.

The model based on only clinical variables had significantly (p< 0.001) poorer discrimination for all outcomes with a variation from c-index 0.58 for stroke prediction to 0.69 for the prediction of hospitalization for HF and was thus consistently outperformed by any of the biomarker models. **(Fig 7).** Application of random survival forest (RF) analyses showed similar results but slightly lower c-indices for all outcomes, especially for outcomes with a lower number of events.

## Discussion

In the present study we have established and validated a novel multiplex quantitative protein panel aimed at improvement of risk stratification, decision support, and treatment precision in patients with CVD. Based on prior screening projects in patients with CAD and AF, 21 proteins were included in our custom designed quantitative PEA panel, CVD-21 tool, for the simultaneous quantitative measurements of these proteins in 1 μL of plasma and a complete analysis of the CVD-21 panel takes 24 hours including preparation of the results. The assays in the CVD-21 tool provided accurate detection and quantification of 20 out of 21 proteins, the exception being the too low sensitivity for troponin I. In the nested case-cohort design including 4224 patients with chronic CAD the evaluation of the discrimination of the risk of future events indicated that the CVD-21 tool together with hs-TnT conveyed similar improvement of biomarkers-based risk stratification compared to clinical variables as multiple biomarkers analyzed by conventional immunoassays. Beyond hs-TnT and NT-proBNP, MMP-12, U-PAR, IL-6, VEGF-D, SCF, ADM, REN, and TFF3 were the most important prognostic biomarkers and might therefore be included in the further development of this type of tool for potential clinical use.

In the CVD-21 panel we have included a number of proteins, which alone or in combinations with other risk markers in the tool are useful for monitoring treatment and risk prediction for future cardiovascular events and adverse drug effects in patients with CAD and AF. The composition of the protein biomarkers in the CVD-21 panel constitutes a unique assembly of biomarkers since they provide protein signatures and prognostic disease patterns covering non-fatal ischemic events (MI, stroke, HF), bleeding during anticoagulant treatment, inflammation and residual inflammatory activity and cardiovascular death, at the same time. The CVD-21 tool, based on multiple biomarkers reflecting multiple organ functions, also provides connections between different cardiovascular disorders in a novel way. Thus, selected biomarkers might help to phenotype patients with CVD into different sub-types of disease and further improve personalized treatment strategies [16, 32, 33].

Approaches using multiplex protein analyses have the potential to identify biomarker profiles to further the understanding of the underlying biology of diseases. Revealing protein functions, complex protein regulatory networks, and interplay between proteins might improve risk stratification and uncover drug-target interactions and drug-target disease mechanisms. The proteins included in the CVD-21 panel reflect a large array of pathophysiological processes. Key biomarkers for CVD events were NT-proBNP, IL-6, and GDF-15 confirming previously reported associations using single marker analyses. The inflammatory marker IL-6 has also been shown to identify CAD patients with so-called residual inflammatory activity, and IL-6 concentrations decrease significantly during targeting of inflammation using a focused cytokine inhibition [6]. In addition to these conventional proteins, the CVD-21 tool constitutes a number of potential novel biomarkers with specific pathways and biological functions with associations to different outcomes as exemplified below. Beyond GDF-15, we have previously shown that osteoprotegerin (OPG), OPN, fibroblast growth factor 23 (FGF-23), and TFF3 are important biomarkers for prediction of bleeding in patients with CAD or AF [21, 34, 35]. The phosphaturic hormone FGF-23 links calcium–phosphate metabolism with left-ventricular dysfunction [36]. Moreover, FGF-23 alone or in combination with NT-proBNP provides identification of patients with undiagnosed silent AF [37, 38]. NT-proBNP, ST2 and ADM have recently been suggested to convey risk prediction for HF and seem to be clinically useful [39]. TFF3, a member of the Trefoil family, is suggested to be a possible risk marker of cardiovascular events, i.e HF [40, 41]. VEGF-D is a secreted glycoprotein that regulates lymphangiogenesis, angiogenesis, fibrogenesis, and apoptosis by binding vascular endothelial growth factor receptors, VEGFR-2 and VEGFR-3 ([42] and references therein). VEGF-D and *VEGFD* genetic variants were recently shown to be independently associated with cardiovascular death in acute as well as chronic CAD [43, 44]. These data may indicate a causal role of VEGF-D in cardiovascular diseases. Finally, SCF, a key growth factor for several types of stem and progenitor cells has been attributed important roles in vasculogenesis and tissue repair [45]. There is clinical support for a potential role of SCF in CVD, including lower risk of MI with high levels [46]. In our validation study this was confirmed since SCF was associated with reduction of risk of all outcomes (**Fig 6A–6C and S3 Fig**).

Selected novel biomarkers in the CVD-21 panel, such as MMP-12, U-PAR, VEGF-D, FGF-23, SCF, ADM, REN, and TFF3 are candidates to proceed with in basic and clinical studies. Whether these new biomarkers in CVD, and their pathways, alone or in combinations might further the understanding of disease pathophysiology, identify novel drug targets, help in development of new drugs, and improve risk prediction and precision medicine, however, need additional in-depth studies.

The proteomic assays in the CVD-21 tool are using the PEA technology in which oligonucleotide-labeled antibody pairs bind their respective antigen in the sample. An important asset of PEA in comparison with other multiplex techniques is the lack of cross-reactivity since the PEA probes are designed for pair-wise hybridization and detection of the antigen that requires double recognition of two specific primers to give a signal [13]. In a recent head-to-head comparison between high-throughput proteomics platforms, the PEA technique was shown to have more reliable target specificity compared to aptamer-based affinity analysis [16]. The CVD-21 tool constitutes a step to the next generation of a specific quantitative PEA-panel including technology to analyze low-abundance as well as high-abundance protein and a pg/ml readout. We also found the quantitative measurements of biomarkers using the CVD-21 tool to have a good agreement with some established single markers assays with correlation coefficients between 0.79–0.87, as shown for the GDF-15 and NT-proBNP assays. These results were also in agreement with our previous comparisons between gold standard targeted immunoassays and PEA NPX values [47].

In the clinical setting an absolute quantification of biomarkers is demanded which enables the direct comparison of data across different laboratories and studies. The CVD-21 PEA employs a microfluidic qPCR technique that assays all the biomarkers simultaneously and converts the signals into protein concentrations in pg/ml units using a standard curve model (see Methods). This differs from regular Olink Target 96 panels, which rely on relative quantification and do not provide data in standard units and also need bridge samples for inter-study comparisons. With the solid normalization and absolute quantification procedure performed for each sample and biomarker in the CVD-21 tool, all users will obtain comparable concentrations of the included biomarkers, and results can be easily compared between different patients. Thus, analyses of biomarkers included in the CVD-21 tool, except for troponin I, are comparable with and may provide an alternative to established single biomarker assays. It is also possible to substitute the 1 μL plasma sample used in a traditional PEA-panel with a 1.2 μm punch of dried blood sample collected on paper [48, 49]. This opens up new possibilities for home sampling that would match well with a centralized tool like CVD-21 for clinical settings and also for population-based screenings. This would allow the combination of the high-throughput and cost-effective PEA technique with specialized hospital clinical methods in order to find patients with CVD at an early stage and thereby improved treatment strategies.

To study the prognostic importance and incremental value of the CVD-21 tool we compared seven multiplex risk prediction models with different combinations of variables, including one model with only clinical variables concerning the 1351 MACE and 296 HF events recorded during 3 years follow-up in the 13,164 patients in the STABILITY biomarker substudy. Harrell´s c-indices were of the same magnitude for all the models with biomarkers including hs-TnT regardless of whether based on CVD-21 or conventional biomarker analyses. The evaluation identified a number of proteins, i.e. MMP-12, U-PAR, Il-6, VEGF-D, SCF, ADM, REN, and TFF3, that provided important prognostic information beyond the established biomarkers hs-TnT and NT-proBNP. These results indicate that the CVD-21 tool might be an alternative tool for improved biomarker-based risk stratification beyond clinical factors and conventionally measured proteins, e.g hs-TnT and NT-proBNP, in patients with CVD. Our results also emphasize and confirm that the addition of biomarkers improves precision in prognostication and the understanding of the reasons for future events in patients with CVD and provide important information beyond that of the traditional clinical risk factors [50].

### Strengths and limitations of the study

The development and validation of the CVD-21 tool form a “road-map” for establishment of multiplex biomarker instruments for decision support in a variety of disorders. The first step of major importance was the selection of proteins based on data from large clinical studies and the screening of 368 biomarkers using conventional immunoassays and four PEA-panels. In the next step, careful analytical validations of the new quantitative PEA-panel were performed. In the final step, the discriminating ability of the tool was analyzed in a large case-control cohort of patients with CCS. A further strength of this clinical validation was that two different statistical methods were employed, a more conventional method and a machine learning technique. The random survival forest algorithm allows for a non-linear association and complex interactions of the variables. A major advantage of the CVD-21 tool is that in one single step, only one low-volume sample is needed for quantification of 21 biomarkers providing complementary prognostic and possible pathophysiological implications.

The very large STABILITY trial with global recruitment and the cohort used in the CVD-21 validation study might not be fully representative of all patients with CCS. However, the STABILITY population is representative of a global population of patients with CCS at increased risk of complications because of the presence of cardiovascular risk factors or co-morbidities under treatment with optimal secondary prevention and cared for by regular outpatient visits at a cardiologist. The present study therefore investigates the importance of simultaneous information on the quantitative levels of 21 proteins and possible pathways that are associated with a residual risk for ischemic events in this setting.

A limitation of the CVD-21 tool was the assay for troponin I which was not sensitive enough with most of the values in the validation study below LLOQ. This limitation of the assay is probably mainly due to a ten-fold predilution of the samples and to the sensitivity and specificity of the used antibodies and PEA probes.

## Conclusions

A novel quantitative multiplex protein panel to support improved precision medicine in CVD was established and clinically validated. The CVD-21 quantitative PEA tool encompassed high specificity and sensitivity and allows low- and high-abundance proteins to be measured simultaneously in one step using only a single small blood sample. The assays in the CVD-21 tool provided accurate quantification of 20 out of 21 proteins, the exception being the too low sensitivity for troponin I. In our nested case-cohort design including 4224 patients with CCS the CVD-21 tool combined with hs-TnT conveyed similar improvement of biomarker-based risk stratification compared to clinical variables and hs-TnT as the utilization of several individual biomarkers analyzed by conventional immunoassays. The CVD-21 panel also contributed specific prognostic information from novel biomarkers that indicate protein signatures and pathophysiological pathways that might further the understanding of the underlying biology of CVD.

## Supporting information

S1 Checklist STROBE statement—checklist of items that should be included in reports of *cohort studies*. (DOCX)

S1 File Reagents for the CVD-21 tool. (DOCX)

S1 Fig Results from linearity study of four assays in the CVD-21 panel. Samples with high endogenous concentrations were diluted with samples with low endogenous concentrations at different ratios and quantified. Relative error (%) was determined for each intermediate data point as measured concentration–theoretical concentration/theoretical concentration x 100. The figure shows measured and theoretical concentrations. Bars indicate standard deviation and the percentage indicate accuracy by each point. All samples were analyzed in triplicate in 3 experiments. Abbreviations: IL6 (interleukin-6), TRAIL-R2 (tumor necrosis factor (TNF)-related apoptosis-inducing ligand 2), U-PAR (soluble urokinase-type plasminogen activator receptor), NT-proBNP (N-terminal prohormone of natriuretic peptide).
(TIF)

S2 Fig Plot of spearman correlations between all biomarkers measured by conventional immunoassays and by quantitative PEA of the biomarkers in the CVD-21 panel. The analyses are performed in the random subset of patients i.e. no enrichment of cases. ECL assays are called lab. Abbreviations: ADM (adrenomedullin), CHI3L1 (chitinase-3 like protein, also called YKL-40 (heparin -and chitin-binding glycoprotein), FGF23 (fibroblast growth factor 23), GDF-15 (growth differentiation factor 15), HGF (hepatocyte growth factor), IL-6 (interleukin-6), TIM- 1/KIM-1 (T-cell immunoglobulin and mucin domain-containing protein), MMP12 (metalloproteinase-12), NT-proBNP (N-terminal prohormone of natriuretic peptide), OPG (osteoprotegerin), OPN (osteopontin), Ren (renin), SCF (stem cell factor), SPON-1 (spondin-1), ST2 (suppression of tumorogenicity), TFF3 (trefoil factor 3), TRAIL-R2 (tumor necrosis factor (TNF)-related apoptosis-inducing ligand 2), Trop I (troponin I), U-PAR (soluble urokinase-type plasminogen activator receptor), VEGF-D (vascular endothelial growth factor -D).
(TIF)

S3 Fig Forest plot for the predictors from the ABC CVD-21 model for the outcome CV-death or hospitalization for heart failure. Variables marked with * are modelled using a four knot spline and has a significant overall effect (the confidence interval could overlap 1). Abbreviations: ADM (adrenomedullin), CHI3L1 (chitinase-3 like protein, also called YKL-40 (heparin -and chitin-binding glycoprotein), FGF23 (fibroblast growth factor 23), GDF-15 (growth differentiation factor 15), HGF (hepatocyte growth factor), IL-6 (interleukin-6), TIM- 1/KIM-1 (T-cell immunoglobulin and mucin domain-containing protein), MMP12 (metalloproteinase-12), NT-proBNP (N-terminal prohormone of natriuretic peptide), OPG (osteoprotegerin), OPN (osteopontin), Ren (renin), SCF (stem cell factor), SPON-1 (spondin-1), ST2 (suppression of tumorogenicity), TFF3 (trefoil factor 3), TRAIL-R2 (tumor necrosis factor (TNF)-related apoptosis-inducing ligand 2), Trop I (troponin I), U-PAR (soluble urokinase-type plasminogen activator receptor), VEGF-D (vascular endothelial growth factor -D).
(PNG)

S1 Table The biomarkers included in the CVD-21 panel and in which 96-plex PEA panel that they are included in. (DOCX)

S2 Table Analysis summary of sensitivity parameters for all assays in the CVD-21 panel. (DOCX)

S3 Table The table shows results from all relative error as average CV%, maximal absolute accuracy percent and average accuracy percent. (DOCX)

S4 Table Plasma samples from the STABILITY trial that were below LLOQ and above ULOQ of the CVD-21 assays. (DOCX)

S5 Table Mean and median accuracy and intra-CV of QC1 and QC2 for the 28 runs of 56 plates in the clinical validation study. (DOCX)

S6 Table Reference values of QC1 and QC2 for the biomarker assays included in the CVD-21 panel. (DOCX)

S7 Table Baseline characteristics in patients with or without (A) major adverse cardiovascular events (MACE) and major coronary events (MCE), or (B) cardiovascular (CV) death or hospitalization for heart failure (HF hosp) or myocardial infarction (MI) in the STABILITY validation study. (DOCX)

S8 Table Descriptives of biomarkers in patients with or without (A) MCE or MACE, or (B) cardiovascular (CV) death or hospitalization for heart failure (HF hosp) or myocardial infarction (MI) in the STABILITY validation study. (DOCX)
