# Supplementary material for: Development and validation of a quantitative Proximity Extension Assay instrument with 21 proteins associated with cardiovascular risk (CVD-21)
Source: PLoS One. 2023 Nov 14;18(11):e0293465. doi: 10.1371/journal.pone.0293465 (PMC10645335; doi:10.1371/journal.pone.0293465)
Supplement: S1 File — (DOCX) [file pone.0293465.s002.docx]

**S1 Material and methods**

**Reagents for the CVD-21 tool**

**Buffers, enzymes, and consumables**

All buffers and enzymes used in the study were from Olink Proteomics: Calibrator Neg (art. no. 44001), Sample Diluent (art. no. 44006), Incubation Solution (art. no. 84369), Detection Solution (art. no. 84315), PEA Enhancer (art. no. 84370), Focus Primer plate (art. no. 84371), PEA Enzyme (art. no. 84303), PCR Polymerase (art. no. 84304) Detection Enzyme (art. no. 84305), 192.24 Dynamic Array™ IFC for Gene Expression art. no. 10962), Sample Diluent (Product No.: SD-001).

**Antibodies and probes**

Twenty-one protein marker assays were developed and combined with three internal control assays (Incubation Control, Extension Control, and Detection Control). The selected biomarker signature included both high and low-abundant proteins. To combine assays for high-abundant proteins that normally require more dilution of the samples with those for lower abundant proteins, the dynamic range for the high-abundant assays must be shifted by optimizations (**S1 Table)**. All samples in the CVD-21 panel were prediluted 1:10 in sample diluent before analysis.

**Calibrators**

Recombinant antigens were chosen to develop calibrator samples to use for normalization and absolute quantification. Three different calibrator samples were developed (Cal High, Cal Middle, Cal Low). Their individual concentrations were chosen to be within the dynamic range of the standard curve and with 4-fold dilutions between. The concentrations for the calibrator high spanned between 0.5-400 pg/ml and 4-fold and 16-fold dilutions were performed to generate the mid and low calibrator samples respectively.

**Preparation of quality controls**

In the *Scandinavian multicenter trial FRISC-II*, which compared the benefits of an early invasive procedure over an early noninvasive strategy and a 90-day prolonged treatment with a low-molecular-weight heparin (dalteparin) versus placebo, 3498 patients were included.[1] Whole blood and plasma samples were obtained at study inclusion and stored at −80°C until analysis. EDTA plasma samples of 200 μL from each of 30 patients randomized to placebo treatment were pooled and used as quality control (QC1) in the CVD-21 tool. The aliquots were stored at -80^o^C until analysis.

Twelve EDTA plasma samples from *healthy individuals* without any medication were collected at the University Hospital, Uppsala, Sweden. 500 μL plasma from each individual was pooled and used as quality control (QC2) in the CVD-21 tool. The aliquots were stored at -80^o^C until analysis.

The reference values for all assays in the CVD-21 panel for QC1 and QC2 were analyzed by the CVD-21 tool in triplicate from nine plates (**S6 Table)**.

**Reference**

1. FRagmin Fast Revascularisation during In Stability in Coronary artery disease (FRISC II) Investigators. Long-term low-molecular-mass heparin in unstable coronary-artery disease: FRISC II prospective randomised multicentre study. FRagmin and Fast Revascularisation during InStability in Coronary artery disease. Investigators. Lancet. 1999;354(9180):701-7.
