# Supplementary figures and images for: Development and validation of a quantitative Proximity Extension Assay instrument with 21 proteins associated with cardiovascular risk (CVD-21)
Source: PLoS One. 2023 Nov 14;18(11):e0293465. doi: 10.1371/journal.pone.0293465 (PMC10645335; doi:10.1371/journal.pone.0293465)

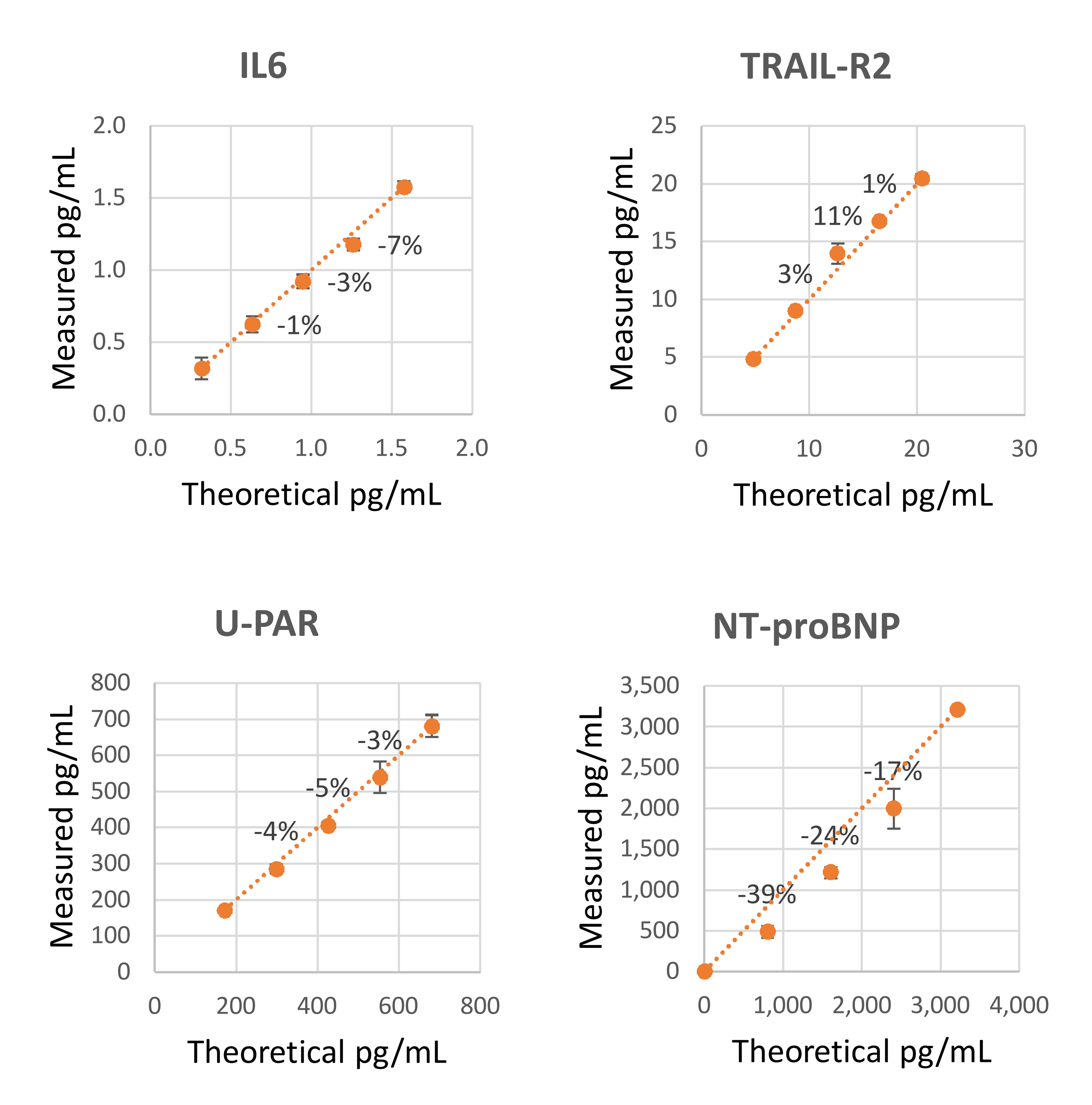

Supplement: S1 Fig — Samples with high endogenous concentrations were diluted with samples with low endogenous concentrations at different ratios and quantified. Relative error (%) was determined for each intermediate data point as measured concentration–theoretical concentration/theoretical concentration x 100. The figure shows measured and theoretical concentrations. Bars indicate standard deviation and the percentage indicate accuracy by each point. All samples were analyzed in triplicate in 3 experiments. Abbreviations: IL6 (interleukin-6), TRAIL-R2 (tumor necrosis factor (TNF)-related apoptosis-inducing ligand 2), U-PAR (soluble urokinase-type plasminogen activator receptor), NT-proBNP (N-terminal prohormone of natriuretic peptide). (TIF) [file pone.0293465.s003.tif]

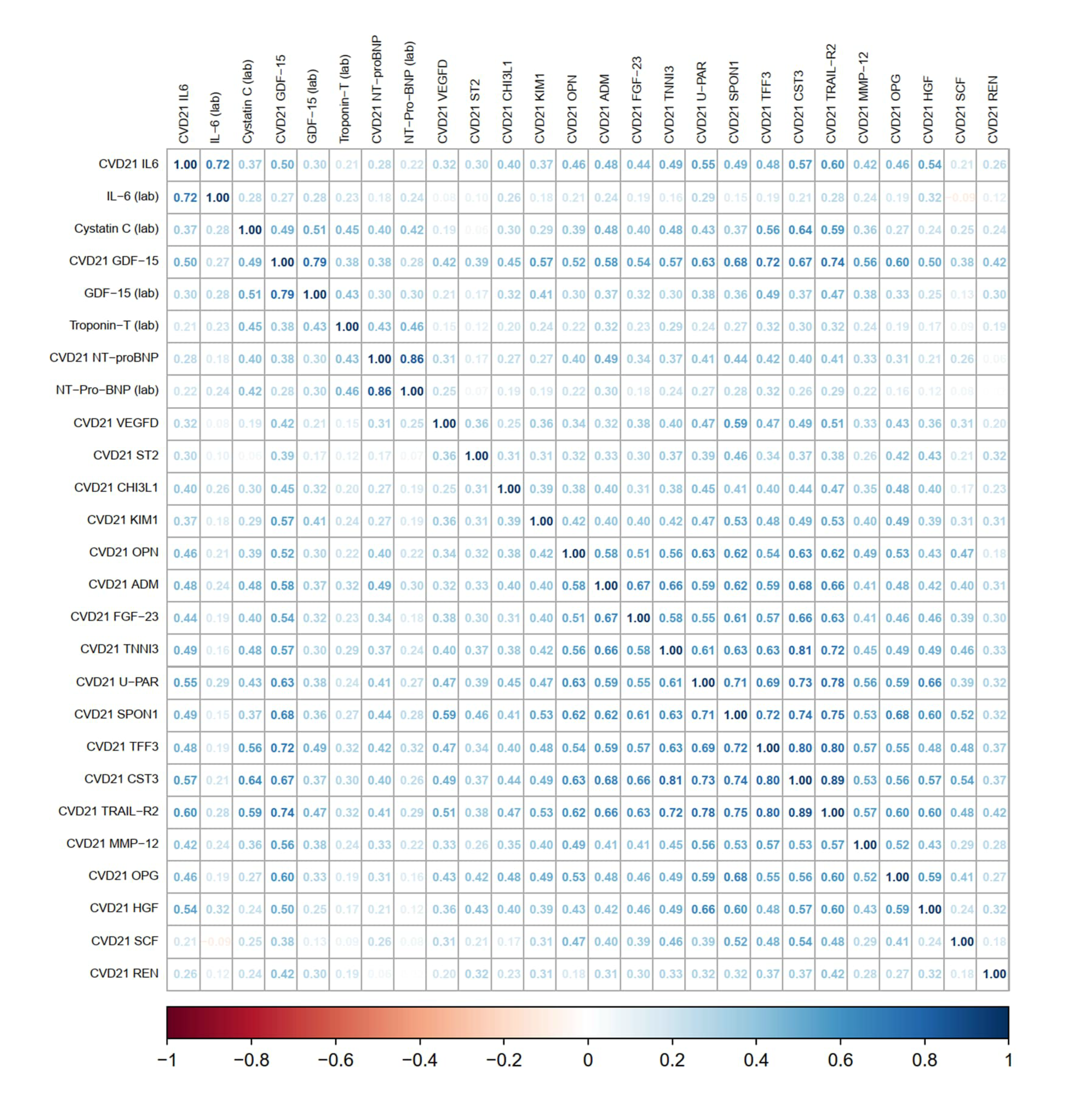

Supplement: S2 Fig — The analyses are performed in the random subset of patients i.e. no enrichment of cases. ECL assays are called lab. Abbreviations: ADM (adrenomedullin), CHI3L1 (chitinase-3 like protein, also called YKL-40 (heparin -and chitin-binding glycoprotein), FGF23 (fibroblast growth factor 23), GDF-15 (growth differentiation factor 15), HGF (hepatocyte growth factor), IL-6 (interleukin-6), TIM- 1/KIM-1 (T-cell immunoglobulin and mucin domain-containing protein), MMP12 (metalloproteinase-12), NT-proBNP (N-terminal prohormone of natriuretic peptide), OPG (osteoprotegerin), OPN (osteopontin), Ren (renin), SCF (stem cell factor), SPON-1 (spondin-1), ST2 (suppression of tumorogenicity), TFF3 (trefoil factor 3), TRAIL-R2 (tumor necrosis factor (TNF)-related apoptosis-inducing ligand 2), Trop I (troponin I), U-PAR (soluble urokinase-type plasminogen activator receptor), VEGF-D (vascular endothelial growth factor -D). (TIF) [file pone.0293465.s004.tif]

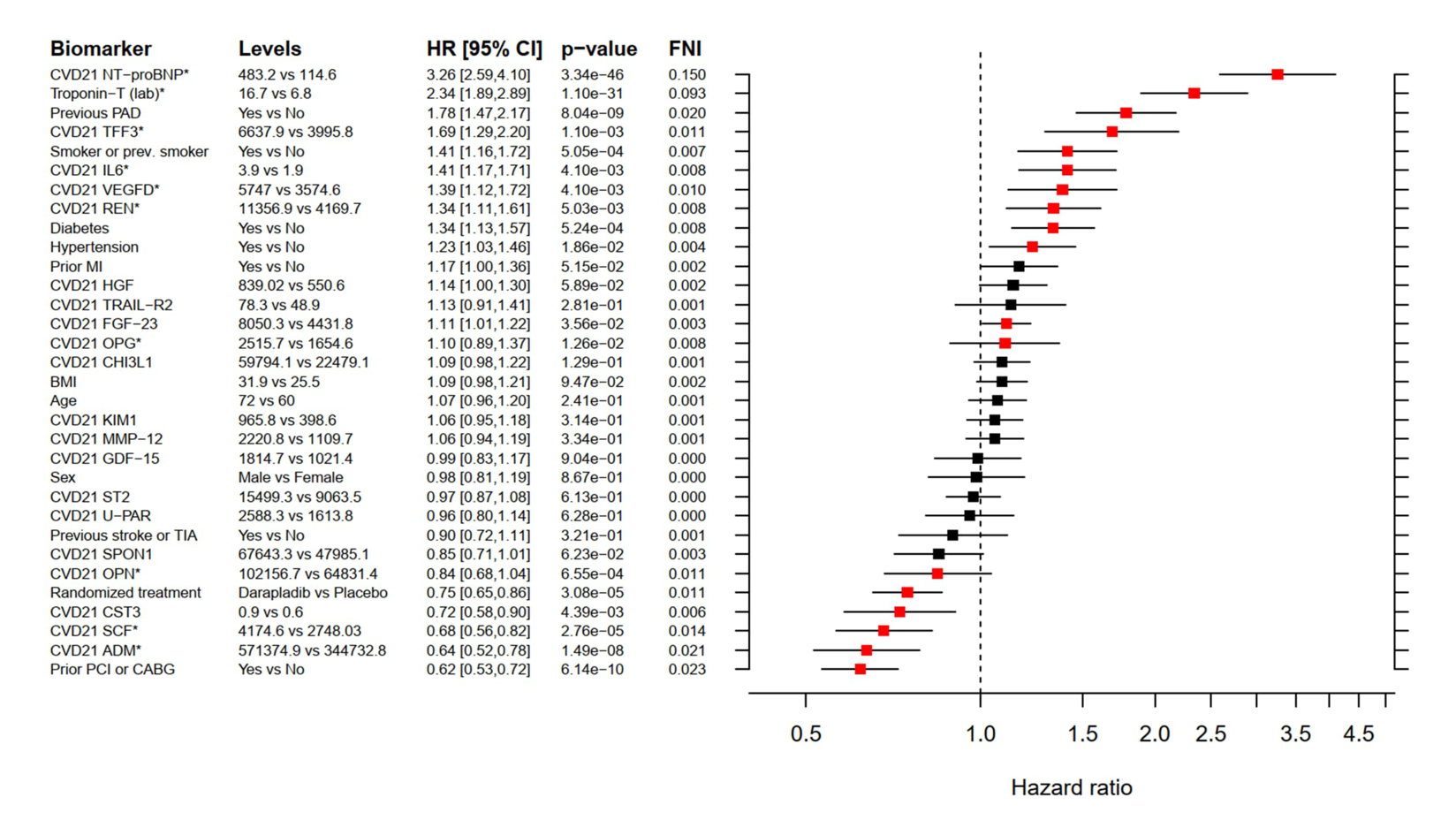

Supplement: S3 Fig — Variables marked with * are modelled using a four knot spline and has a significant overall effect (the confidence interval could overlap 1). Abbreviations: ADM (adrenomedullin), CHI3L1 (chitinase-3 like protein, also called YKL-40 (heparin -and chitin-binding glycoprotein), FGF23 (fibroblast growth factor 23), GDF-15 (growth differentiation factor 15), HGF (hepatocyte growth factor), IL-6 (interleukin-6), TIM- 1/KIM-1 (T-cell immunoglobulin and mucin domain-containing protein), MMP12 (metalloproteinase-12), NT-proBNP (N-terminal prohormone of natriuretic peptide), OPG (osteoprotegerin), OPN (osteopontin), Ren (renin), SCF (stem cell factor), SPON-1 (spondin-1), ST2 (suppression of tumorogenicity), TFF3 (trefoil factor 3), TRAIL-R2 (tumor necrosis factor (TNF)-related apoptosis-inducing ligand 2), Trop I (troponin I), U-PAR (soluble urokinase-type plasminogen activator receptor), VEGF-D (vascular endothelial growth factor -D). (PNG) [file pone.0293465.s005.png]
