# Supplementary material for: Development and validation of a quantitative Proximity Extension Assay instrument with 21 proteins associated with cardiovascular risk (CVD-21)
Source: PLoS One. 2023 Nov 14;18(11):e0293465. doi: 10.1371/journal.pone.0293465 (PMC10645335; doi:10.1371/journal.pone.0293465)
Supplement: S1 Table — (DOCX) [file pone.0293465.s006.docx]

| **Biomarker** | **96-plex panel** | **96-plex panel dilution** | **Uniprot ID** | **Optimized assays for dynamic range** |
| --- | --- | --- | --- | --- |
| Trop I | Organ Damage | 1:1 | P19429 |  |
| NT-proBNP | Metabolism | 1:10 | - |  |
| Cystatin C | Cardiometabolic | 1:2025 | P01034 | Yes |
| SCF | Inflammation | 1:1 | P21583 |  |
| FGF23 | Inflammation | 1:1 | Q9GZV9 |  |
| HGF | Inflammation/OncII | 1:1 | P14210 |  |
| TIM-1/KIM-1 | CVDI/CVDII | 1:1 | Q96D42 |  |
| IL-6 | CVD II | 1:1 | P05231 |  |
| MMP-12 | CVD II | 1:1 | P39900 |  |
| Ren | CVD II | 1:1 | P00797 |  |
| VEGF-D | CVD II | 1:1 | O43915 |  |
| TRAIL-R2 | CVD II | 1:1 | O14763 |  |
| ADM | CVD II | 1:1 | P35318 |  |
| GDF-15 | CVD III | 1:100 | Q99988 | Yes |
| OPN | CVD III | 1:100 | P10451 | Yes |
| OPG | CVD III/Inflammation | 1:100/1:1 | O00300 |  |
| SPON-1 | CVD III | 1:100 | Q9HCB6 | Yes |
| ST2 | CVD III | 1:100 | Q01638 | Yes |
| TFF3 | CVD III | 1:100 | Q07654 | Yes |
| U-PAR | CVD III | 1:100 | Q03405 | Yes |
| CHI3L1 | CVD III | 1:100 | P36222 | Yes |

Some of the assays were optimized for expected dynamic range of the target protein concentrations in clinical samples. All plasma samples analyzed by the CVD-21 panel are prediluted 1:10.

Abbreviations: ADM (adrenomedullin), CHI3L1 (chitinase-3 like protein, also called YKL-40 (heparin -and chitin-binding glycoprotein), FGF23 (fibroblast growth factor 23), GDF-15 (growth differentiation factor 15), HGF (hepatocyte growth factor), IL-6 (interleukin-6), TIM- 1/KIM-1 (T-cell immunoglobulin and mucin domain-containing protein), MMP12 (metalloproteinase-12), NT-proBNP (N-terminal prohormone of natriuretic peptide), OPG (osteoprotegerin), OPN (osteopontin), Ren (renin), SCF (stem cell factor), SPON-1 (spondin-1), ST2 (suppression of tumorogenicity), TFF3 (trefoil factor 3), TRAIL-R2 (tumor necrosis factor (TNF)-related apoptosis-inducing ligand 2), Trop I (troponin I), U-PAR (soluble urokinase-type plasminogen activator receptor), VEGF-D (vascular endothelial growth factor -D).
