# Supplementary material for: Development and validation of a quantitative Proximity Extension Assay instrument with 21 proteins associated with cardiovascular risk (CVD-21)
Source: PLoS One. 2023 Nov 14;18(11):e0293465. doi: 10.1371/journal.pone.0293465 (PMC10645335; doi:10.1371/journal.pone.0293465)
Supplement: S2 Table — (DOCX) [file pone.0293465.s007.docx]

| **Biomarker** | **LOD** | **LLOQ** | **ULOQ** | **Range** | **HOOK** |
| --- | --- | --- | --- | --- | --- |
| TIM1/KIM1 | 31 | 31 | 31250 | 3.0 | 62500 |
| NT-proBNP | 0.7 | 1.6 | 10273 | 3.8 | 10273 |
| SCF | 3.8 | 15 | 15625 | 3.0 | 62500 |
| VEGF-D | 15 | 61 | 31250 | 2.7 | 250000 |
| suPAR | 0.5 | 0.9 | 7812 | 3.9 | 15625 |
| IL-6 | 0.03 | 0.1 | 920 | 3.9 | 7358 |
| TFF3 | 1.9 | 3.8 | 15625 | 3.6 | 62500 |
| Cystatin C | 161 | 323 | 165159 | 2.7 | 330318 |
| TRAIL-R2 | 0.1 | 0.2 | 3906 | 4.2 | 31250 |
| MMP-12 | 1.9 | 3.8 | 7812 | 3.3 | 125000 |
| ST2 | 7.6 | 15 | 62500 | 3.6 | 500000 |
| OPN | 76 | 305 | 625000 | 3.3 | 1250000 |
| HGF | 1.9 | 7.6 | 7812 | 3.0 | 62500 |
| GDF-15 | 1.7 | 3.4 | 1732 | 2.7 | 55419 |
| Trop I | 0.7 | 19 | 15967 | 2.9 | 15967 |
| OPG | 1.9 | 7.6 | 7812 | 3.0 | 62500 |
| ADM | 488 | 3906 | 1000000 | 2.4 | 4000000 |
| FGF23 | 122 | 244 | 250000 | 3.0 | 500000 |
| SPON-1 | 122 | 244 | 125000 | 2.7 | 1000000 |
| CHI3L | 30.5 | 122 | 250000 | 3.3 | 500000 |
| REN | 3.8 | 15 | 7812 | 2.7 | 250000 |

All values are pg/ml. The dynamic range is log10. LOD = level of detection. LLOQ = lower level of quatification. ULOQ = upper level of quatification.

Abbreviations: ADM (adrenomedullin), CHI3L1 (chitinase-3 like protein, also called YKL-40 (heparin -and chitin-binding glycoprotein), FGF23 (fibroblast growth factor 23), GDF-15 (growth differentiation factor 15), HGF (hepatocyte growth factor), IL-6 (interleukin-6), TIM- 1/KIM-1 (T-cell immunoglobulin and mucin domain-containing protein), MMP12 (metalloproteinase-12), NT-proBNP (N-terminal prohormone of natriuretic peptide), OPG (osteoprotegerin), OPN (osteopontin), Ren (renin), SCF (stem cell factor), SPON-1 (spondin-1), ST2 (suppression of tumorogenicity), TFF3 (trefoil factor 3), TRAIL-R2 (tumor necrosis factor (TNF)-related apoptosis-inducing ligand 2), Trop I (troponin I), U-PAR (soluble urokinase-type plasminogen activator receptor), VEGF-D (vascular endothelial growth factor -D).
