# Supplementary material for: Development and validation of a quantitative Proximity Extension Assay instrument with 21 proteins associated with cardiovascular risk (CVD-21)
Source: PLoS One. 2023 Nov 14;18(11):e0293465. doi: 10.1371/journal.pone.0293465 (PMC10645335; doi:10.1371/journal.pone.0293465)
Supplement: S3 Table — (DOCX) [file pone.0293465.s008.docx]

| **Assay** | **Precision:**  **Average %CV** | **Relative error:**  **Max (ABS) %** | **Relative error: Average %** |
| --- | --- | --- | --- |
| TIM1/KIM1 | 3% | 11% | -1% |
| Trop I (TNNI3) | 9% | 2% | -1% |
| CST3 | 4% | 19% | -7% |
| OPG | 4% | 7% | -1% |
| NT-proBNP | 8% | 39% | -14% |
| TRAIL-R2 | 4% | 14% | 1% |
| ADM | 4% | 14% | -6% |
| SCF | 9% | 6% | 2% |
| MMP-12 | 3% | 7% | 0% |
| FGF23 | 5% | 17% | -3% |
| VEGFD | 5% | 8% | -2% |
| ST2 | 3% | 8% | 0% |
| SPON-1 | 4% | 16% | -7% |
| U-PAR | 4% | 6% | -2% |
| OPN | 5% | 6% | 1% |
| CHI3L1 | 3% | 9% | -1% |
| IL6 | 7% | 13% | 0% |
| HGF | 4% | 8% | 0% |
| REN | 4% | 7% | 0% |
| TFF3 | 3% | 6% | 1% |
| GDF-15 | 3% | 13% | -2% |

Abbreviations: ADM (adrenomedullin), CHI3L1 (chitinase-3 like protein, also called YKL-40 (heparin -and chitin-binding glycoprotein), FGF23 (fibroblast growth factor 23), GDF-15 (growth differentiation factor 15), HGF (hepatocyte growth factor), IL-6 (interleukin-6), TIM- 1/KIM-1 (T-cell immunoglobulin and mucin domain-containing protein), MMP12 (metalloproteinase-12), NT-proBNP (N-terminal prohormone of natriuretic peptide), OPG (osteoprotegerin), OPN (osteopontin), Ren (renin), SCF (stem cell factor), SPON-1 (spondin-1), ST2 (suppression of tumorogenicity), TFF3 (trefoil factor 3), TRAIL-R2 (tumor necrosis factor (TNF)-related apoptosis-inducing ligand 2), Trop I (troponin I), U-PAR (soluble urokinase-type plasminogen activator receptor), VEGF-D (vascular endothelial growth factor -D).
