# Supplementary material for: Development and validation of a quantitative Proximity Extension Assay instrument with 21 proteins associated with cardiovascular risk (CVD-21)
Source: PLoS One. 2023 Nov 14;18(11):e0293465. doi: 10.1371/journal.pone.0293465 (PMC10645335; doi:10.1371/journal.pone.0293465)
Supplement: S4 Table — (DOCX) [file pone.0293465.s009.docx]

| **Biomarker** | **Number** | **LLOQ** | **N below  LLOQ** | **Percent below  LLOQ** | **ULOQ** | **N above  ULOQ** | **Percent above  ULOQ** |
| --- | --- | --- | --- | --- | --- | --- | --- |
| ADM | 4224 | 3906 | 60 | 1.4 | 1000000 | 0 | 0 |
| CHI3L1 | 4224 | 122 | 2 | 0.0 | 250000 | 0 | 0 |
| CST3 | 4224 | 323 | 2 | 0.0 | 165159 | 25 | 0.6 |
| FGF-23 | 4224 | 244 | 167 | 4.0 | 250000 | 0 | 0 |
| GDF-15 | 4224 | 3.4 | 2 | 0.0 | 1732 | 0 | 0 |
| HGF | 4224 | 7.6 | 2 | 0.0 | 7812 | 0 | 0 |
| IL6 | 4224 | 0.1 | 244 | 5.8 | 920 | 0 | 0 |
| MMP-12 | 4224 | 3.8 | 1 | 0.0 | 7812 | 0 | 0 |
| NT-proBNP | 4224 | 1.6 | 21 | 0.5 | 10273 | 0 | 0 |
| OPG | 4224 | 7.6 | 1 | 0.0 | 7812 | 0 | 0 |
| OPN | 4224 | 305 | 2 | 0.0 | 625000 | 0 | 0 |
| REN | 4224 | 15 | 2 | 0.0 | 7812 | 1 | 0 |
| SCF | 4224 | 15 | 2 | 0.0 | 15625.0 | 0 | 0 |
| SPON1 | 4224 | 244.1 | 2 | 0.0 | 125000.0 | 0 | 0 |
| ST2 | 4224 | 15 | 2 | 0.0 | 62500 | 0 | 0 |
| TFF3 | 4224 | 3.8 | 1 | 0.0 | 15625 | 0 | 0 |
| TIM1/KIM1 | 4224 | 31 | 499 | 11.8 | 31250 | 0 | 0 |
| TNNI3 | 4224 | 19 | 4100 | 97.1 | 15967 | 0 | 0 |
| TRAIL-R2 | 4224 | 0.2 | 2 | 0.0 | 3906 | 0 | 0 |
| U-PAR | 4224 | 0.9 | 1 | 0.0 | 7812 | 0 | 0 |
| VEGFD | 4224 | 61 | 16 | 0.4 | 31250 | 0 | 0 |

All values are pg/ml. LLOQ = lower level of quantification. ULOQ = upper level of quantification.

Abbreviations: ADM (adrenomedullin), CHI3L1 (chitinase-3 like protein, also called YKL-40 (heparin -and chitin-binding glycoprotein), FGF23 (fibroblast growth factor 23), GDF-15 (growth differentiation factor 15), HGF (hepatocyte growth factor), IL-6 (interleukin-6), TIM- 1/KIM-1 (T-cell immunoglobulin and mucin domain-containing protein), MMP12 (metalloproteinase-12), NT-proBNP (N-terminal prohormone of natriuretic peptide), OPG (osteoprotegerin), OPN (osteopontin), Ren (renin), SCF (stem cell factor), SPON-1 (spondin-1), ST2 (suppression of tumorogenicity), TFF3 (trefoil factor 3), TRAIL-R2 (tumor necrosis factor (TNF)-related apoptosis-inducing ligand 2), Trop I (troponin I), U-PAR (soluble urokinase-type plasminogen activator receptor), VEGF-D (vascular endothelial growth factor -D).
