# Supplementary material for: Development and validation of a quantitative Proximity Extension Assay instrument with 21 proteins associated with cardiovascular risk (CVD-21)
Source: PLoS One. 2023 Nov 14;18(11):e0293465. doi: 10.1371/journal.pone.0293465 (PMC10645335; doi:10.1371/journal.pone.0293465)
Supplement: S5 Table — (DOCX) [file pone.0293465.s010.docx]

|  |  | **QC1** |  |  |  |  |  | **QC2** |  |  |  |  |
| --- | --- | --- | --- | --- | --- | --- | --- | --- | --- | --- | --- | --- |
|  |  | **Accuracy** | |  | **Intra-CV** | |  | **Accuracy** | |  | **Intra-CV** | |
| **Assay** |  | **Mean** | **Median** |  | **Mean** | **Median** |  | **Mean** | **Median** |  | **Mean** | **Median** |
| ADM |  | 100.2 | 100.6 |  | 11.1 | 10.1 |  | 101.9 | 97.5 |  | 11.1 | 10.8 |
| CHI3L1 |  | 96.6 | 92.1 |  | 13.4 | 12.4 |  | 96.7 | 93.1 |  | 14.0 | 11.4 |
| CST3 |  | 96.7 | 95.9 |  | 13.1 | 11.2 |  | 99.7 | 94.3 |  | 13.3 | 11.9 |
| FGF23 |  | 96.3 | 95.1 |  | 13.6 | 12.3 |  | 98.3 | 92.8 |  | 18.8 | 16.3 |
| GDF-15 |  | 96.1 | 92.8 |  | 12.4 | 10.2 |  | 98.5 | 94.0 |  | 12.2 | 9.4 |
| HGF |  | 98.9 | 98.1 |  | 11.6 | 10.3 |  | 99.3 | 96.3 |  | 13.0 | 11.6 |
| IL6 |  | 95.9 | 93.5 |  | 18.5 | 17.2 |  | 82.5 | 77.5 |  | 29.0 | 24.7 |
| KIM1 |  | 98.1 | 94.5 |  | 14.5 | 13.4 |  | 88.5 | 82.6 |  | 18.0 | 14.6 |
| MMP-12 |  | 96.4 | 92.3 |  | 12.5 | 10.8 |  | 97.8 | 94.6 |  | 13.3 | 11.5 |
| NT-proBNP |  | 93.3 | 92.3 |  | 13.0 | 10.6 |  | 96.8 | 97.1 |  | 19.7 | 19.5 |
| OPG |  | 93.0 | 90.2 |  | 12.7 | 11.0 |  | 94.6 | 91.4 |  | 12.4 | 9.9 |
| OPN |  | 99.5 | 98.4 |  | 12.1 | 11.3 |  | 100.7 | 98.0 |  | 13.3 | 11.2 |
| REN |  | 97.5 | 94.9 |  | 12.5 | 11.0 |  | 98.9 | 94.0 |  | 12.6 | 10.8 |
| SCF |  | 97.7 | 95.9 |  | 10.6 | 9.0 |  | 97.6 | 94.4 |  | 10.7 | 9.4 |
| SPON1 |  | 94.8 | 93.7 |  | 12.2 | 10.1 |  | 96.4 | 93.9 |  | 12.0 | 10.1 |
| ST2 |  | 98.8 | 97.7 |  | 13.2 | 12.1 |  | 99.3 | 95.1 |  | 13.0 | 11.3 |
| TFF3 |  | 92.2 | 88.6 |  | 12.9 | 9.8 |  | 94.1 | 90.0 |  | 13.0 | 12.0 |
| TNNI3 |  | N/A | N/A |  | 20.8 | 19.9 |  | N/A | N/A |  | 26.3 | 22.7 |
| TRAIL-R2 |  | 95.1 | 93.7 |  | 14.8 | 12.6 |  | 96.5 | 93.1 |  | 15.4 | 11.9 |
| U-PAR |  | 95.1 | 93.0 |  | 13.1 | 11.1 |  | 95.4 | 90.7 |  | 13.1 | 11.6 |
| VEGFD |  | 92.9 | 92.3 |  | 11.9 | 12.3 |  | 93.7 | 93.3 |  | 12.7 | 11.2 |

All values are percentage. QC1 reference values for accuracy are based on patients with cardiovascular disease and QC2 reference values are for healthy blood donors.

Abbreviations: ADM (adrenomedullin), CHI3L1 (chitinase-3 like protein, also called YKL-40 (heparin -and chitin-binding glycoprotein), FGF23 (fibroblast growth factor 23), GDF-15 (growth differentiation factor 15), HGF (hepatocyte growth factor), IL-6 (interleukin-6), TIM- 1/KIM-1 (T-cell immunoglobulin and mucin domain-containing protein), MMP12 (metalloproteinase-12), NT-proBNP (N-terminal prohormone of natriuretic peptide), OPG (osteoprotegerin), OPN (osteopontin), Ren (renin), SCF (stem cell factor), SPON-1 (spondin-1), ST2 (suppression of tumorogenicity), TFF3 (trefoil factor 3), TRAIL-R2 (tumor necrosis factor (TNF)-related apoptosis-inducing ligand 2), Trop I (troponin I), U-PAR (soluble urokinase-type plasminogen activator receptor), VEGF-D (vascular endothelial growth factor -D).
