# Supplementary material for: Development and validation of a quantitative Proximity Extension Assay instrument with 21 proteins associated with cardiovascular risk (CVD-21)
Source: PLoS One. 2023 Nov 14;18(11):e0293465. doi: 10.1371/journal.pone.0293465 (PMC10645335; doi:10.1371/journal.pone.0293465)
Supplement: S6 Table — (DOCX) [file pone.0293465.s011.docx]

| **Assay** | **Reference value, pg/ml QC1** | **Reference value, pg/ml QC2** |
| --- | --- | --- |
| ADM | 469790 | 245970 |
| CHI3L1 | 27350 | 11810 |
| CST3 | 702400 | 418360 |
| FGF-23 | 8070 | 3500 |
| GDF-15 | 1390 | 610 |
| HGF | 1050 | 390 |
| IL-6 | 4.2 | 1.3 |
| KIM1 | 720 | 300 |
| MMP-12 | 2380 | 930 |
| NT-proBNP | 373 | 50 |
| OPG | 2170 | 1540 |
| OPN | 82200 | 59260 |
| REN | 5110 | 3040 |
| SCF | 3350 | 3290 |
| SPON1 | 59240 | 40730 |
| ST2 | 12090 | 11710 |
| TFF3 | 5190 | 2820 |
| TNNI3 | <LLOQ | <LLOQ |
| TRAIL-R2 | 60 | 31 |
| U-PAR | 1950 | 1200 |
| VEGF-D | 4160 | 3440 |
|  |  |  |

The quality control (QC) 1 was a pool of plasma samples from patients with acute CAD. QC2 was a pool of plasma samples from healthy individuals. The reference values were analyzed by the CVD-21 tool in triplicate from 9 plates.

Abbreviations: ADM (adrenomedullin), CHI3L1 (chitinase-3 like protein, also called YKL-40 (heparin -and chitin-binding glycoprotein), FGF23 (fibroblast growth factor 23), GDF-15 (growth differentiation factor 15), HGF (hepatocyte growth factor), IL-6 (interleukin-6), TIM- 1/KIM-1 (T-cell immunoglobulin and mucin domain-containing protein), MMP12 (metalloproteinase-12), NT-proBNP (N-terminal prohormone of natriuretic peptide), OPG (osteoprotegerin), OPN (osteopontin), Ren (renin), SCF (stem cell factor), SPON-1 (spondin-1), ST2 (suppression of tumorogenicity), TFF3 (trefoil factor 3), TRAIL-R2 (tumor necrosis factor (TNF)-related apoptosis-inducing ligand 2), Trop I (troponin I), U-PAR (soluble urokinase-type plasminogen activator receptor), VEGF

D (vascular endothelial growth factor -D).
