# Supplementary material for: Development and validation of a quantitative Proximity Extension Assay instrument with 21 proteins associated with cardiovascular risk (CVD-21)
Source: PLoS One. 2023 Nov 14;18(11):e0293465. doi: 10.1371/journal.pone.0293465 (PMC10645335; doi:10.1371/journal.pone.0293465)
Supplement: S7 Table — (DOCX) [file pone.0293465.s012.docx]

Table S7A

| **Baseline characteristics** | **MCE, no events**  **(N=2726** | **MCE, events**  **(N=1163)** | **MACE, no events**  **(N=2720)** | **MACE, events**  **(N=1351)** |
| --- | --- | --- | --- | --- |
| **Age** | 65 (59 - 71) | 67 (60 - 73) | 65 (59 - 71) | 67 (60 – 73) |
| **Sex: Male** | 82 (2235) | 82 (953) | 82 (2234) | 82 (1105) |
| **BMI** | 28 (25 - 32) | 29 (26 - 32) | 28 (25 - 32) | 28 (26 - 32) |
| **Weight (kg)** | 82 (72 - 94) | 82 (72 - 94) | 82 (72 - 94) | 84 (72 - 94) |
| **Smoker or prev. smoker** | 19 (525) | 22 (254) | 19 (527) | 21 (282) |
| **Hypertension** | 70 (1907) | 75 (876) | 70 (1903) | 76 (1023) |
| **Diabetes** | 38 (1023) | 48 (561) | 37 (1017) | 48 (643) |
| **Prior MI** | 59 (1601) | 64 (740) | 58 (1591) | 63 (854) |
| **Prior PCI or CABG** | 75 (2054) | 71 (825) | 76 (2060) | 70 (947) |
| **Previous stroke or TIA** | 8 (225) | 11 (129) | 8 (222) | 12 (165) |
| **Previous PAD** | 8 (208) | 14 (165) | 8 (206) | 14 (187) |
| **Prior multivessel CHD** | 13 (355) | 18 (206) | 13 (357) | 17 (234) |
| **Polyvascular disease** | 14 (395) | 23 (270) | 14 (394) | 23 (316) |
| **Randomized treatment: Darapladib** | 52 (1411) | 47 (542) | 52 (1404) | 48 (647) |
| **aspirin** | 92 (2521) | 91 (1055) | 92 (2513) | 91 (1224) |
| **Baseline P2Y_12_inhibitor** | 33 (899) | 36 (421) | 33 ((906) | 34 (460) |
| **Baseline β-blockade** | 78 (2139) | 80 (930) | 79 (2141) | 78 (1059) |
| **Baseline statins** | 97 (2649) | 97 (1126) | 97 (2642) | 97 (1309) |
| **Baseline ACE or ARB** | 76 (2071) | 79 (921) | 76 (2066) | 79 (1073) |

m (a – b) represents median (Q1 – Q3).

p (n) represent percentage (frequency). Percentages computed by group.

Table S7B

| **Baseline characteristics** | **CV death or HF hosp, no events (N=2825)** | **CV death or HF hosp, events (N=831)** | **MI, no events (N=2884)** | **MI, events  (N=652)** |
| --- | --- | --- | --- | --- |
| **Age (years)** | 65 (59 - 71) | 61.5 (69 - 75) | 65 (59 - 71) | 66 (59 - 73) |
| **Sex: Male** | 82 (2319) | 82 (683) | 82 (2366) | 80 (522) |
| **BMI (kg/m^2^)** | 28 (26 - 32) | 29 (26 - 33) | 28.1 (26 - 32) | 29.0 (26 - 33) |
| **Weight (kg)** | 82 (72 - 94) | 83 (71 - 94) | 82 (72 - 94) | 84 (73 - 95) |
| **Smoker or prev. smoker** | 20 (553) | 19 (159) | 20 (564) | 22 (142) |
| **Hypertension** | 70 (1972) | 80 (661) | 70 (2025) | 74 (484) |
| **Diabetes** | 37 (1054) | 55 (458) | 38 (1103) | 46 (298) |
| **Prior MI** | 59 (1653) | 65 (542) | 59 (1696) | 62 (403) |
| **Prior PCI or CABG** | 76 (2139) | 65 (544) | 75 (2163) | 78 (509) |
| **Previous stroke or TIA** | 8 (238) | 13 (105) | 8 (239) | 11 (74) |
| **Previous PAD** | 8 (215) | 16 (137) | 8 (229) | 13 (85) |
| **Prior multivessel CHD** | 13 (367) | 20 (164) | 14 (393) | 15 (100) |
| **Polyvascular disease** | 15 (411) | 27 (223) | 15 (426) | 22 (144) |
| **TRTCD : 2** | 52 (1462) | 47 (391) | 51 (1482) | 48 (311) |
| **Prior aspirin** | 92 (2610) | 89 (736) | 92 (2663) | 92 (600) |
| **Prior P2Y_12_inhibitor** | 34 (952) | 31 (259) | 33 (963) | 38 (251) |
| **Prior Betablocker** | 79 (2220) | 81 (669) | 79 (2265) | 79 (518) |
| **Prior statins** | 97 (2744) | 97 (810) | 97 (2805) | 96 (624) |
| **Prior ACEi/ARB** | 76 (2144) | 81 (669) | 76 (2191) | 78 (511) |

m (a – b) represents median (Q1 – Q3).

p (n) represent percentage (frequency). Percentages computed by group.

Abbreviations: MI (myocardial infarction), PCI (Percutaneous Coronary Intervention), CABG (Coronary Artery Bypass Graft), TIA (transient ischemic attack), PAD (peripheral artery disease), CHD (coronary heart disease), ACEi (angiotensin-converting enzyme inhibitor), ARB (angiotensin receptor blocker).
