# Supplementary material for: Development and validation of a quantitative Proximity Extension Assay instrument with 21 proteins associated with cardiovascular risk (CVD-21)
Source: PLoS One. 2023 Nov 14;18(11):e0293465. doi: 10.1371/journal.pone.0293465 (PMC10645335; doi:10.1371/journal.pone.0293465)
Supplement: S8 Table — (DOCX) [file pone.0293465.s013.docx]

Table S8A

| **Biomarker** | **MCE**  **no events**  **N=2726** | **MCE**  **events**  **N= 1163** | **MACE**  **no events**  **N= 2720** | **MACE**  **events**  **N=1351** |
| --- | --- | --- | --- | --- |
| KIM1 | 583 (382- 898) | 696 (456-1090) | 577 (381-889) | 701 (462- 1108) |
| Troponin I | 0.062 (0.045-0.083) | 0.071 (0.051- 0.100) | 0.061 (0.045-0.082) | 0.070 (0.052-0.09) |
| CST3 | 0.71 (0.59- 0.89) | 0.80 (0.64 -1.03) | 0.71 (0.59-0.89) | 0.80 (0.64-1.02) |
| OPG | 1977 (1622- 2420) | 2138 (1723-2659) | 1972 (1612 2417) | 2148 (1758-2658) |
| NT-proBNP | 186 (98-364) | 334 (149-758) | 184 (98-363) | 330 (149-751) |
| TRAIL-R2 | 58 (47-73) | 66 (52-87) | 58 (47-73) | 67 (52-87) |
| ADM | 426680 (337916-536516) | 485462 (369733- 628047) | 425806 (337659 -535553) | 481277 (370617-621278) |
| SCF | 3400 (2757-4179) | 3313 (2631- 4109) | 3391 (2753-4171) | 3338 (2682-4119) |
| MMP-12 | 1440 (1056-2034) | 1746 (1235-2584) | 1435 (1057-2029) | 1750 (1230-2612) |
| FGF-23 | 5681 (4294-7532) | 6497 (4733 -9066) | 5675 (4278-7481) | 6476 (4742-8997) |
| VEGF-D | 4285 (3453-5413) | 4802 (3811-6285) | 4274 (3450-5411) | 4776 (3816-6238) |
| ST2 | 11549 (8917-15008) | 12615 (9664-16422) | 11519 (8891-15000) | 12621 (9742-16267) |
| SPON 1 | 55129 (4666-65050) | 59696 (50107-71771) | 55025 (46522-64930) | 59828 (50218-71278) |
| U-PAR | 1923 (1566-2423) | 2223 (1698-2882) | 1917 (1559-2409) | 2223 (1701-2870) |
| OPN | 79257 (63977-97819) | 85175 (67400-108669) | 79006 (63876-97569) | 85480 (68050-109909) |
| CHI3L1 | 32933 (21182-54687) | 39199 (24886-67468) | 32824 (21122-54435) | 39813 (24948-66471) |
| IL-6 | 2.5 (1.8-3.6) | 3.1 (2.1-4.6) | 2.5 (1.8-3.6) | 3.1 (2.1-4.6) |
| HGF | 651 (536-808) | 718 (581-885) | 649 (533-808) | 719 (584-878) |
| REN | 6199 (4042-10324) | 7427 (4370-13700) | 6126 (4033-10227) | 7368 (4370-13427) |
| GDF-15 | 1265 (978-1671) | 1473 (1136-2056) | 1258 (975-1661) | 1483 (1147-2051) |
| Cystatin C (lab) | 0.99 (0.86-1.16) | 1.10 (0.92-1.35) | 0.99 (0.86-1.16) | 1.09 (0.92-1.35) |
| NT-proBNP (lab) | 172 (82-340) | 355 (142-850) | 170 (82-339) | 350 (139-831) |
| Troponin-T (lab) | 9.2 (6.3-13.9) | 13.5 (8.7-21.9) | 9.2 (6.3-13.8) | 13.4 (8.6-21.8) |
| GDF-15 (lab) | 1243 (907-1754) | 1540 (1081-2318) | 1227 (904-1738) | 1555 (1090-2327) |
| IL-6 (lab) | 2.1 (1.4-3.1) | 2.7 (1.7-4.3) | 2.1 (1.4-3.2) | 2.6 (1.7-4.2) |
| TFF3 | 4836 (3867-6143) | 5641 (4252-7575) | 4813 (3853-6128) | 5595 (4252-7528) |

M (a – b) represents median (Q1 – Q3). All values are ng/L except for cystatin C (mg/L) and troponin I (ng/ml).

**Table S8B**

| **Biomarker** | **CV death or HF hosp,**  **no events (N=2825)** | **CV death or HF hosp,**  **Events (N=831)** | **MI, no events (N=2884)** | **MI, events  (N=652)** |
| --- | --- | --- | --- | --- |
| KIM1 | 581.4 (381.0 - 888.7) | 799.8 (505.5 - 1259.2) | 586.4 (384.9 - 906.2) | 674.6 (429.8 – 1030.0) |
| TNNI3 | 0.062 (0.045 - 0.083) | 0.079 (0.055 - 0.11) | 0.062 (0.045 - 0.084) | 0.068 (0.049 - 0.096) |
| CST3 | 0.71 (0.59 - 0.89) | 0.88 (0.69 - 1.13) | 0.72 (0.59 - 0.90) | 0.78 (0.61 - 0.98) |
| OPG | 1977.6 (1617.4 – 2413.0) | 2317.5 (1842.0 - 2918.4) | 1987.4 (1622.4 – 2435.0) | 2070.3 (1699.8 - 2568.8) |
| NT-proBNP | 184.5 (97.9 - 362.0) | 524.5 (251.5 - 1185.9) | 190.0 (99.3 - 380.0) | 263.4 (129.2 - 549.8) |
| TRAIL-R2 | 58.1 (47.3 - 73.0) | 73.4 (56.9 - 97.2) | 58.4 (47.5 - 73.8) | 63.4 (50.5 - 83.5) |
| ADM | 428350.4 (338103.1 - 538493.7) | 526254.5 (392716.3 - 687761.5) | 429894.0 (338293.9 - 542047.8) | 470235.6 (364551.7 - 596643.1) |
| SCF | 3387.2 (2754.5 – 4158.0) | 3377.8 (2715.3 - 4193.4) | 3381.8 (2745.0 - 4171.4) | 3320.1 (2609.9 - 4105.0) |
| MMP-12 | 1443.6 (1060.6 – 2050.0) | 1925.4 (1361.7 - 2828.7) | 1450.3 (1063.4 - 2063.7) | 1686.3 (1175.5 - 2543.0) |
| FGF23 | 5690.6 (4299.8 - 7520.2) | 7060.0 (5069.3 - 10369.1) | 5703.9 (4312.9 - 7569.8) | 6377.9 (4585.8 - 8971.1) |
| VEGFD | 4274.3 (3450.6 - 5396.8) | 5349.2 (4261.4 - 7253.1) | 4328.1 (3473.9 - 5479.6) | 4596.5 (3693.2 - 5896.2) |
| ST2 | 11533.0 (8905.4 - 15010.2) | 13164.0 (10325.9 - 17886.5) | 11601.7 (8924.3 - 15085.9) | 12464.6 (9513.2 - 15741.6) |
| SPON1 | 55111.9 (46576.7 - 64959.8) | 64413.6 (53495.7 - 77613.9) | 55267.2 (46821.0 - 65454.8) | 58206.3 (48957.7 - 69038.0) |
| U-PAR | 1928.3 (1566.0 - 2423.6) | 2384.4 (1878.3 - 3113.0) | 1934.9 (1572.1 - 2452.6) | 2148.7 (1645.6 - 2822.5) |
| OPN | 79395.0 (64055.4 - 98230.7) | 90085.9 (69744.8 - 116721.9) | 79637.8 (64041.5 - 98050.2) | 83387.5 (66832.4 - 106111.6) |
| CHI3L1 | 32815.9 (21180.3 - 54432.1) | 46994.7 (29375.1 – 82875.0) | 33471.1 (21426.7 - 55070.7) | 35390.0 (23381.0 - 60294.5) |
| IL6 | 2.5 (1.8 - 3.6) | 3.4 (2.4 - 5.3) | 2.5 (1.8 - 3.7) | 3.0 (2.1 - 4.4) |
| HGF | 651.5 (535.5 - 808.7) | 766.6 (627.6 – 949.0) | 655.5 (537.4 - 813.5) | 701.5 (562.2 - 858.9) |
| REN | 6238.6 (4037.2 - 10297.8) | 8616.2 (4784.4 - 15308.9) | 6261.4 (4082.5 - 10509.1) | 7308.5 (4226.8 - 13028.8) |
| TFF3 | 4820.7 (3848.1 - 6155.9) | 6338.9 (4813.4 - 8522.8) | 4866.6 (3883.4 - 6224.3) | 5240.2 (4057.3 - 6962.5) |
| GDF-15 | 1266.0 (977.3 - 1672.6) | 1669.4 (1252.1 - 2348.7) | 1273.1 (982.3 - 1686.4) | 1403.9 (1100.7 - 1949.1) |
| Cystatin C (lab) | 1.0 (0.9 - 1.2) | 1.2 (1.0 - 1.5) | 1.0 (0.9 - 1.2) | 1.1 (0.9 - 1.3) |
| NT-Pro-BNP (lab) | 171.0 (83.0 - 342.5) | 612.5 (269.8 - 1340.8) | 178.5 (86.0 - 371.0) | 260.5 (113.8 - 591.0) |
| Troponin-T (lab) | 9.2 (6.3 - 13.9) | 17.2 (11.3 - 26.8) | 9.4 (6.3 - 14.4) | 11.8 (7.9 - 19.0) |
| GDF-15 (lab) | 1235.0 (909.5 - 1763.5) | 1789.0 (1256.3 - 2779.5) | 1255.0 (917.0 - 1786.0) | 1488.0 (1035.0 - 2147.5) |
| IL-6 (lab) | 2.1 (1.4 - 3.2) | 3.0 (2.0 - 4.9) | 2.1 (1.4 - 3.2) | 2.5 (1.7 - 3.9) |

M (a – b) represents median (Q1 – Q3). All values are ng/L except for cystatin C (mg/L) and troponin I (ng/ml).

Abbreviations: ADM (adrenomedullin), CHI3L1 (chitinase-3 like protein, also called YKL-40 (heparin -and chitin-binding glycoprotein), FGF23 (fibroblast growth factor 23), GDF-15 (growth differentiation factor 15), HGF (hepatocyte growth factor), IL-6 (interleukin-6), TIM- 1/KIM-1 (T-cell immunoglobulin and mucin domain-containing protein), MMP12 (metalloproteinase-12), NT-proBNP (N-terminal prohormone of natriuretic peptide), OPG (osteoprotegerin), OPN (osteopontin), Ren (renin), SCF (stem cell factor), SPON-1 (spondin-1), ST2 (suppression of tumorogenicity), TFF3 (trefoil factor 3), TRAIL-R2 (tumor necrosis factor (TNF)-related apoptosis-inducing ligand 2), Trop I (troponin I), U-PAR (soluble urokinase-type plasminogen activator receptor), VEGF-D (vascular endothelial growth factor -D).
